# Bone morphogenetic proteins 4 and 7 increase human white and brown adipocyte thermogenic capacity

**DOI:** 10.1172/jci.insight.194140

**Published:** 2026-03-12

**Authors:** Kelly T. Long, Cheryl Cero, Sahara L. Ali, Nhuquynh Nguyen, Adrienne R. Guarnieri, Ju Hee Kim, Young Jae Bahn, Jurgen Heymann, Jonathan M. Dreyfuss, Sushil G. Rane, Yu-Hua Tseng, Aaron M. Cypess

**Affiliations:** 1 Diabetes, Endocrinology, and Obesity Branch, National Institute of Diabetes and Digestive and Kidney Diseases (NIDDK), NIH, Bethesda, Maryland, USA.; 2 Bioinformatics & Biostatistics Core, Joslin Diabetes Center, and; 3 Integrative Physiology and Metabolism Section, Joslin Diabetes Center, Harvard Medical School, Boston, Massachusetts, USA.

**Keywords:** Endocrinology, Metabolism, Adipose tissue, Bioenergetics, Signal transduction

## Abstract

Adipocytes exist along a functional spectrum: white adipocytes are energy storing, and brown adipocytes have thermogenic capacity such that activation may counteract obesity-related disease. In between are UCP1-expressing beige adipocytes, which can transition between these two energetic states. We previously showed that bone morphogenetic protein 7 (BMP7), a member of the TGF-β superfamily, enables differentiation of brown preadipocytes to mature thermogenic cells. To see whether immortalized, clonal human white and brown preadipocytes (hWAs and hBAs, respectively) would become more thermogenic in response to BMP exposure, we treated them with BMP7 or BMP4 for the first 7 days of a 30-day differentiation protocol. In hBAs, absence of either BMP7 or BMP4 led to lower expression of brown-specific markers and oxygen consumption relative to 7 days with either BMP. hWAs treated for 7 days with either BMP did not increase expression of thermogenic protein UCP1 nor induce a brown-like transcription profile. However, BMP-treated hWAs produced adipocytes that had higher basal and drug-induced maximal oxygen consumption, which was UCP1-independent and due substantially to the futile creatine cycle. Our results demonstrate that energetically quiescent hWAs can be pushed into an energy-expending phenotype without transdifferentiation into beige adipocytes, providing a new approach to treat obesity-related metabolic disease.

## Abstract 2

Treating human white adipocytes with specific bone morphogenetic proteins (BMP4 or BMP7) transitions them to distinct “tan” adipocytes with expanded energy expending capabilities.

## Graphical abstract

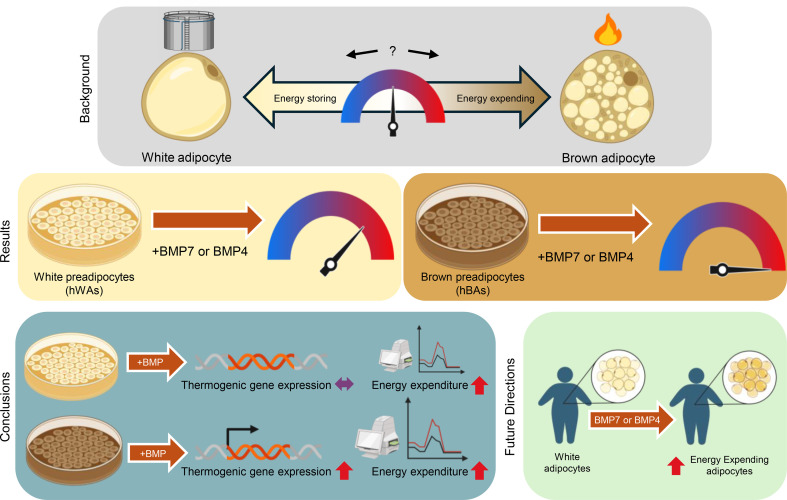

## Introduction

Human adipose tissue is increasingly understood as a complex organ that plays a central role in regulation of metabolic homeostasis. White adipose tissue (WAT) acts as an energy reservoir and monitoring station for energy balance, secreting paracrine and endocrine factors to regulate other metabolic tissues (1). WAT also contributes to the complications associated with increasing obesity rates and related metabolic diseases. Brown adipose tissue (BAT) and the functionally related beige adipocytes (2) are energy-expending cells that mediate non-shivering thermogenesis primarily through mitochondrial uncoupling protein 1 (UCP1) (3). The discovery of BAT in adult humans, which was formerly thought to only be present in infants, has reignited interest in BAT activation as a therapeutic strategy to treat obesity (4).

Despite interest in harnessing BAT to treat metabolic disease, the relatively low volume of BAT in humans (0.1% to 0.5% of total body mass) compared with mice (2% to 5% of total body mass) makes it such that activation of BAT thermogenesis may not be sufficient to provide the excess energy expenditure required for weight loss and other metabolic benefits (5). Beige adipocytes are a class of inducible UCP1-expressing cells. The origins of human beige adipocytes remain incompletely defined, but they are thought to derive from some combination of (a) de novo differentiation from a population of beige precursors and (b) conversion (transdifferentiation) from mature white adipocytes to beige adipocytes (2, 6, 7). “Beiging” of adipocytes is of great interest due to the large volume of white adipose tissue (WAT) present in the human body. Converting even a fraction of this total volume from energy-storing white fat to more energetically active fat may yield substantial metabolic benefits.

Cold exposure and adrenergic stimulation are known methods of inducing a beige response, but the patient discomfort associated with continuous cold exposure and the side effects of tachycardia and hypertension associated with adrenergic stimulation require further exploration of alternative viable approaches (8). Bone morphogenetic protein 7 (BMP7) is a member of the TGF-β superfamily, which has been found to activate brown adipogenesis and increase energy expenditure in mice (9). In humans, BMP7 strongly upregulated thermogenesis in brown adipocytes derived from the human neck area and induced “beiging” in a subcutaneous adipose tissue cell line (10–12).

Given the strong evidence of the importance of BMP7 facilitating thermogenesis, we hypothesized that treating human white preadipocytes (hWAs) (13) with BMP7 prior to differentiation would induce a shift to higher energy expenditure. Conversely, excluding BMP7 exposure of human brown preadipocytes (hBAs) would lead to a reduction in thermogenic capacity. To determine whether the BMP7 effects were more of a general reflection of TGF-β signaling during hBA and hWA differentiation, we assessed whether BMP4, a related member, could induce similar effects. We found that a 7-day treatment with either BMP7 or BMP4 are important in hBAs for proper maturation and in hWAs for increasing energy-expending capacity.

## Results

### Short-term but not long-term BMP7 increases the thermogenic function of hBAs.

**Figure 1:**
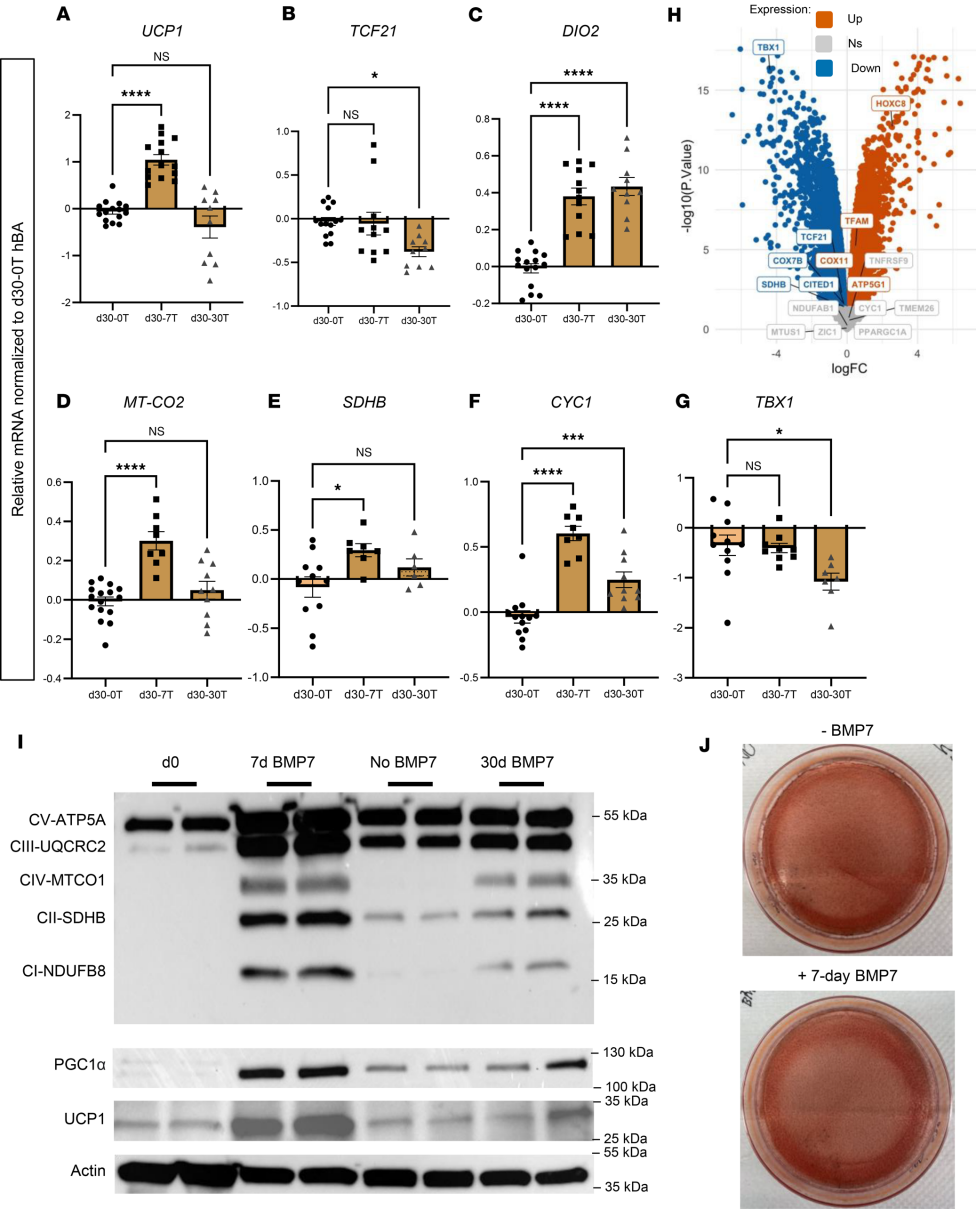
Omitting BMP7 treatment in hBAs decreased brown and thermogenic markers. qPCR analysis of hBAs at 30 days of differentiation without BMP7 treatment (light brown bars), 30 days of differentiation with 7 days of BMP7 treatment (dark brown bars), and 30 days of differentiation with 30 days of BMP7 treatment (checkered dark brown bars). The mRNA expression profiles of (**A**) thermogenic markers, (**B**) white adipocyte markers, (**C**) brown adipocyte markers, (**D**) mitochondrial markers, (**E** and **F**) electron transport chain markers, and (**G**) beige adipocyte markers (*P < 0.05; ***P < 0.001; ****P < 0.0001). (*n* = 8–16 replicates). d30-0T, d30-7T, and d30-30T refer to expression levels after 30 days of differentiation while being exposed to 0, 7, or 30 days of treatment with BMP7. (**H**) Volcano plot depicting the relationship between –log_2_ (*P* value) and log_2_ (fold change) in microarray data, contrasting the treated (pre-BMP7 and BMP7) with the control (no BMP7) groups. Blue text and points indicate upregulated genes in the treated condition; orange designates downregulated genes. Gray labels denote nonsignificant genes. (**I**) Immunoblot showing protein levels of OXPHOS proteins, PGC1α, and UCP1 in hBAs. Actin used as the loading control. (**J**) Oil Red O staining of differentiated clonal immortalized brown adipocytes. Statistical analysis for qPCR data was conducted using 1-way ANOVA with Benjamini-Hochberg procedure to correct for multiple comparisons.

Prior studies have demonstrated the importance of BMP7 for differentiation and thermogenic function of hBAs (9). To assess the effects of short-term and continuous BMP7 exposure on the hBA gene expression profile, we treated hBAs undergoing a 30-day differentiation regimen with BMP7 for 7 or 30 days. We evaluated transcriptional markers established to be associated with the following: thermogenesis, white adipocytes, brown adipocytes, mitochondrial function, electron transport chain (ETC), and beige adipocytes (Figure 1, A–G, and Supplemental Figure 1, A–D; supplemental material available online with this article; https://doi.org/10.1172/jci.insight.194140DS1) (2, 7, 13–15). As expected, 7 days of BMP7 exposure significantly upregulated expression of thermogenic *UCP1* (Figure 1A). In addition, 7 days of BMP7 maintained baseline expression of white adipocyte marker *TCF21* (Figure 1B), while increasing brown adipocyte marker *DIO2* (Figure 1C), mitochondrial gene *MT-CO2* (Figure 1D), and ETC genes *SDHB* and *CYC1* (Figure 1, E and F). Beige adipocyte marker *TBX1* was unaffected by 7 days of BMP7 (Figure 1G). To get a perspective on how generalized these findings were, we analyzed unpublished microarray data from a separate set of human brown adipose tissue stromal vascular fraction (hBAT-SVF) cells derived from the deep neck that were differentiated using a related but modified protocol as detailed by Tseng et al. (16). There was a similarly decreased expression of beige adipocyte markers *TCF21*, *CITED1*, and *TBX1* (Figure 1H).

In contrast to 7 days of BMP7, exposure to BMP7 for all 30 days of differentiation did not augment the thermogenic gene expression profile but instead reduced it. There was only a baseline expression of *UCP1* (Figure 1A)*, MT-CO2* (Figure 1D), and *SDHB* (Figure 1E). Additionally, 30-day BMP7 lowered expression of white adipocyte marker *TCF21* (Figure 1B) and beige adipocyte marker *TBX1* (Figure 1G). Neither 7 days nor 30 days of BMP7 affected expression of transcriptional coactivator *PPARGC1A*, but 30 days of BMP7 maintained increased gene expression of brown marker *MTUS1*, mitochondrial marker *TFAM*, and ETC gene *NDUFAB1* (Supplemental Figure 1, A–D). To determine whether these transcriptional changes were reflected at the protein level, we performed immunoblots for ETC proteins, UCP1, and the transcriptional coactivator and regulator of brown fat thermogenesis PGC1α (Figure 1I). We found that 7-day BMP7 upregulated expression of ETC proteins, UCP1, and PGC1α, while expression was lower in 30-day BMP7-treated hBAs (Figure 1I). Although BMP7 affected browning of hBAs, it did not appear to affect lipid droplet accumulation across the 30-day differentiation protocol (Figure 1J). Overall, compared with 7 days, a full 30 days of exposure to BMP7 signaling during hBA differentiation induced an expression profile that was less transcriptionally thermogenic and “brown.”

### BMP7 decreases expression of white adipocyte markers in hWAs without increasing UCP1.

**Figure 2:**
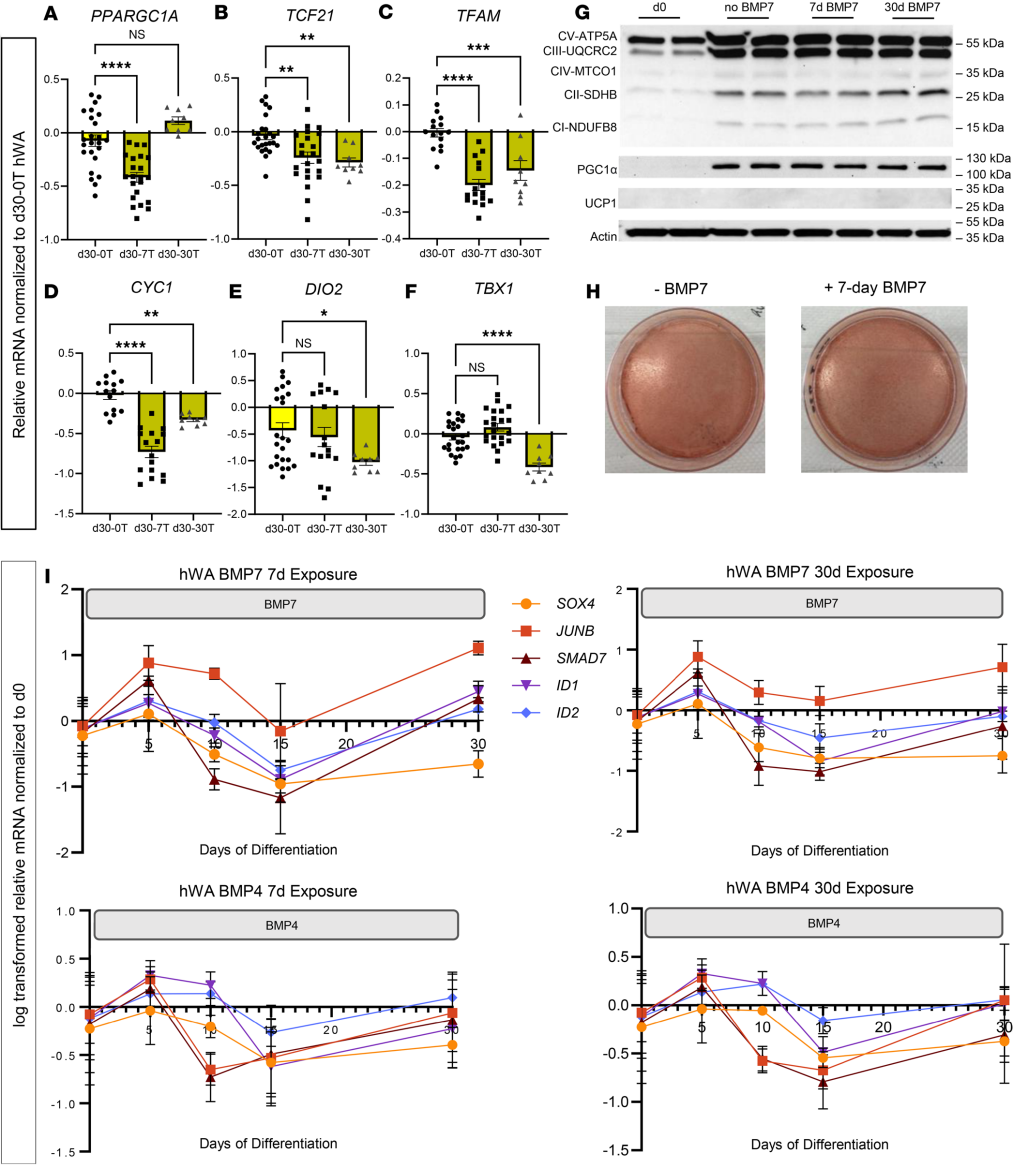
BMP7 treatment decreased TCF21 white adipocyte markers and decreased beige and thermogenic markers in hWAs. qPCR analysis of hWAs at 30 days of differentiation without BMP7 treatment (yellow bars), 30 days of differentiation with 7 days of BMP7 treatment (dark yellow bars), and 30 days of differentiation with 30 days of BMP7 treatment (checkered dark yellow bars). The mRNA expression profiles of (**A**) thermogenic markers, (**B**) white adipocyte markers, (**C**) mitochondrial markers, (**D**) electron transport chain markers, (**E**) brown adipocyte markers, and (**F**) beige adipocyte markers (*P < 0.05; **P < 0.01; ***P < 0.001; ****P < 0.0001). (*n* = 8–24 replicates). d30-0T, d30-7T, and d30-30T refer to expression levels after 30 days of differentiation while being exposed to 0, 7, or 30 days of treatment with BMP7. (**G**) Immunoblot showing protein levels of OXPHOS proteins, PGC1α, and UCP1 in hWAs. Actin used as the loading control. (**H**) Oil Red O staining of differentiated clonal immortalized white adipocytes. (**I**) mRNA expression profiles of TGF-β–associated genes in hWAs treated with BMP7 or BMP4 for 7 or 30 days (indicated by gray box). Statistical analysis for qPCR data was conducted using 1-way ANOVA with Benjamini-Hochberg procedure to correct for multiple comparisons.

Beige adipocytes, a class of inducible thermogenic adipocytes, have been of high interest since it has been suggested that they can be activated via adrenergically mediated “browning” of WAT (2, 17, 18). As such, we hypothesized that BMP7 could also promote “beiging” and thermogenic function in hWAs. The response was more nuanced in that 7-day BMP7 decreased expression of thermogenic marker *PPARGC1A* (Figure 2A), and both 7-day and 30-day BMP7 exposure decreased expression of *TCF21* (Figure 2B), *TFAM* (Figure 2C), and *CYC1* (Figure 2D). We found that 30-day BMP7 decreased expression of *DIO2* (Figure 2E) and *TBX1* (Figure 2F). BMP7 exposure did not affect expression of *ZIC1* (Supplemental Figure 2A), but 30-day exposure did decrease expression of *MTUS1* (Supplemental Figure 2B). Moreover, 7 days and 30 days of BMP7 decreased ETC genes *NDUFAB* and *COX7B* (Supplemental Figure 2, C and D). We also examined the impact of adding BMP7 to hWAs for the last 7 days of differentiation (days 23 to 30). There was no effect on expression of thermogenic marker *PPARGC1A*, white adipocyte maker *TCF21*, mitochondrial marker *TFAM*, ETC marker *CYC1*, brown adipocyte marker *DIO2*, and beige adipocyte marker *TBX1* (Supplemental Figure 3, A–F). Additionally, BMP7 treatment did not affect *BMPR1A* and *BMPR2* gene expression compared with the control group (Supplemental Figure 3, G and H). Taken together, these results suggest that BMP7 exposure during later stages of differentiation does not affect the thermogenic capacity of hWAs. This interpretation supports our initial hypothesis that early exposure to BMP7 is crucial to induce an energy-expending phenotype in hWAs without transdifferentiation into beige adipocytes.

We next determined whether these changes in mRNA levels translated to alterations in corresponding cellular proteins and functional capacity. BMP7 did not affect expression of ETC proteins or PGC1α in the white adipocytes (Figure 2G). As with hBAs, BMP7 did not affect lipid droplet accumulation across differentiation: at the macroscopic level, there was no difference in lipid accumulation when determined via Oil Red O accumulation (Figure 2H). In addition, fluorescence microscopy demonstrated that both the standard and the BMP7-treated hWAs had a similar number of nuclei. However, BMP7-treated hWAs had lower amounts of total neutral lipids, multilocular lipid droplets, and unilocular lipid droplets but exhibited larger multilocular lipid droplets than hWAs (Supplemental Figure 4, A–J). Given that human white adipocytes have predominantly unilocular lipid droplets while brown adipocytes have multilocular lipid droplets (19), these data support the possibility that BMP7-treated hWAs are more brown-like (i.e., thermogenic). Taken together, BMP7 induced in hWAs an expression profile that was less characteristically “white,” but without explicitly promoting “browning” or “beiging.” Rather, these cells appeared to fall elsewhere along the brown-white adipocyte continuum.

We assessed whether variations in BMP receptor (BMPR) levels may explain differences in the pattern of gene expression changes in hBAs and hWAs. BMP7 is known to bind to both the type I receptor BMPR1A and the type II receptor BMPR2 (20). From day 0 of differentiation to day 30, expression of the receptor BMPR1A increased in both hBAs and hWAs, but expression of BMPR2 remained stable (Supplemental Figure 5, A–D). BMPR1A expression was unaffected by BMP7 exposure in hBAs but increased in hWAs with either 7-day or 30-day exposure (Supplemental Figure 5, E and G). BMPR2 expression in hBAs was increased by 30-day BMP7 exposure and increased in hWAs with either 7-day or 30-day BMP7 exposure (Supplemental Figure 5, F and H). Altogether, the dynamic nature of BMPR expression may be consistent with a pattern where the adipocytes became progressively sensitized to BMP7 signaling after an initial exposure period.

### BMP4 induces an expression pattern in hBAs and hWAs similar to BMP7.

**Figure 3:**
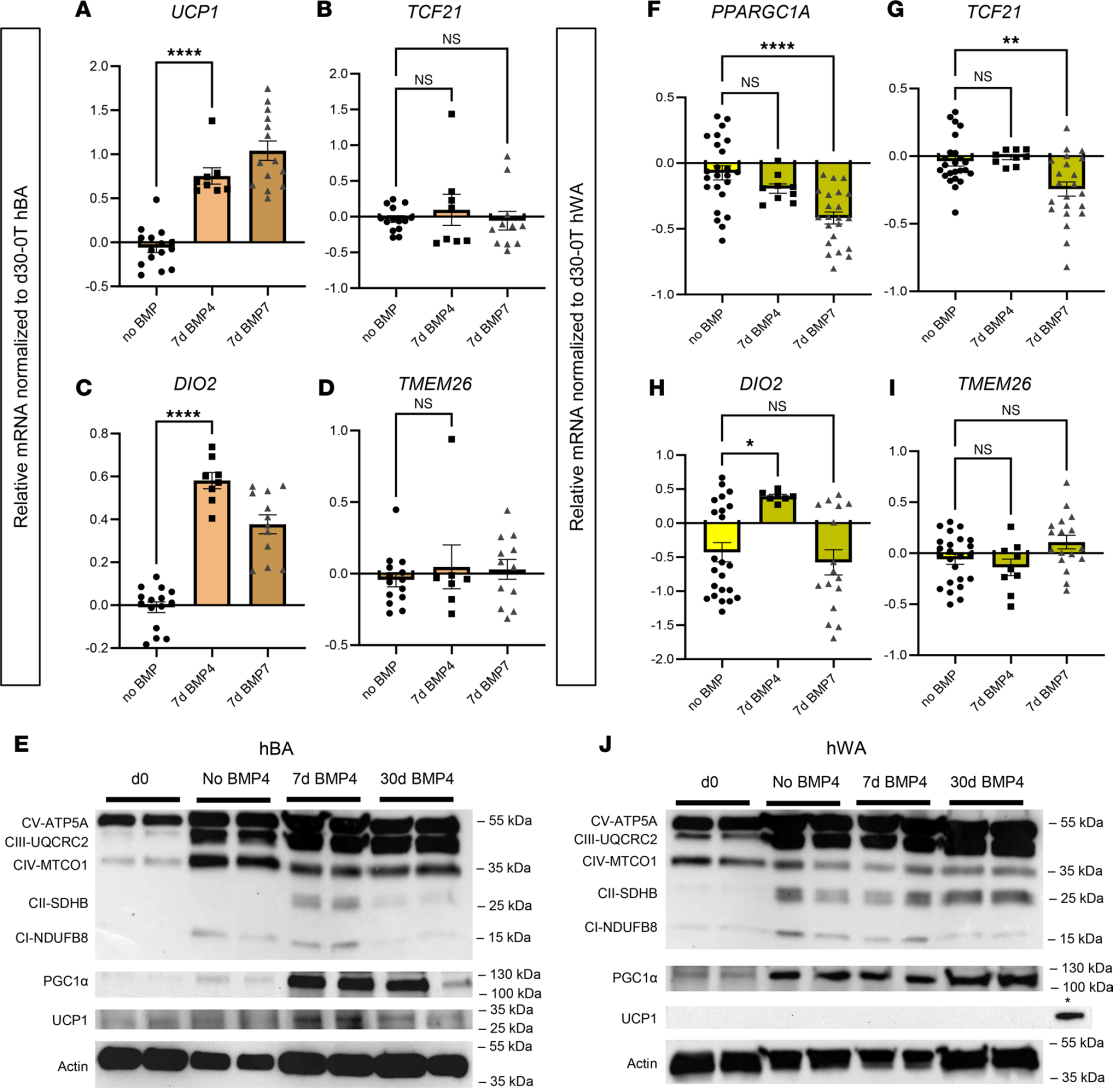
BMP4 treatment of 7 days in hBAs and hWAs induces a gene expression pattern similar to BMP7. qPCR analysis of brown (**A**–**D**) and white (**F**–**I**) adipocytes at 30 days of differentiation with 7 days of no BMP treatment (dark brown bars), 30 days of differentiation with BMP4 treatment (light brown bars), and 30 days of differentiation with 7 days of BMP7 treatment (checkered dark brown bars). The mRNA expression profiles of (**A** and **F**) thermogenic markers, (**B** and **G**) white adipocyte markers, (**C** and **H**) brown adipocyte markers, and (**D** and **I**) beige adipocyte markers (*P < 0.05; **P < 0.01; ****P < 0.0001). (*n* = 8–24 replicates). Each data point shown represents a biological replicate. (**E** and **J**) Immunoblots in hBAs and hWAs, respectively, showing protein levels of OXPHOS proteins and PGC1α. For the hBA blot, the (+) above 7d BMP4 indicates the standard conditions, which is the positive control. In the hWA UCP1 blot, the (*) above rightmost band is the positive control, which was hBAs treated for 7 days with BMP4. Actin used as the loading control. Statistical analysis for qPCR data was conducted using 1-way ANOVA with Benjamini-Hochberg procedure to correct for multiple comparisons.

Amongst the BMP family, BMP7 has been reported as the singular member to induce thermogenic properties in mouse BAs (9). However, in human adipose tissue models, the ability to promote thermogenic transition is seemingly not exclusive to BMP7 (11). BMP4 is another member of the TGF-β superfamily and shares a receptor binding target BMPR1A with BMP7 (20, 21). We therefore hypothesized that BMP4 may also augment the thermogenic function of hBAs, characterized by the upregulation of specific genes associated with thermogenesis through the TGF-β signaling pathway. We assessed expression patterns of TGF-β–associated genes (Figure 2I) alongside markers of adipocyte identity and function (Figure 3, A–D). In hBAs, we found that BMP4 induced a transcriptional profile paralleling that of BMP7, with higher expression of *UCP1* and *DIO2* (Figure 3, A–D). Exposure to BMP4 for 7 or 30 days also increased *MTUS1* similarly to BMP7 (Supplemental Figure 6A). We found that 7-day BMP4 also increased expression of ETC proteins and UCP1, and as was the case with BMP7, this pattern was reversed with 30-day treatment (Figure 3E).

In hWAs, 7-day BMP4 had a distinct effect on gene expression, maintaining basal-level expression of *PPARGC1A* and *TCF21* while increasing expression of brown marker *DIO2* and beige marker *TMEM26* (Figure 3, F–I). Similar to BMP7, BMP4 did not affect expression of *MTUS1* (Supplemental Figure 6B). Unlike with BMP7, 7-day BMP4 increased expression of additional beige markers *TBX1* and *CITED1* (Supplemental Figure 6, C and D). Immunoblots indicated that BMP4 promotes a similar protein expression profile as BMP7 in hWAs (Figure 3, E and J).

Given the overall similar expression profiles of hWAs induced by BMP7 and BMP4, both members of the TGF-β family, we assessed the behavior of the TGF-β intracellular signaling pathway. We conducted time course analysis of the expression of TGF-β–responsive genes (*SOX4*, *JUNB*, *SMAD7*, *ID1*, and *ID2*) across 30 days of differentiation via qPCR (Figure 2I). Treatment of hWAs for 7 days with either BMP7 or BMP4 downregulated TGF-β signaling during the first 15 days of differentiation, followed by recovery to baseline by the end of differentiation at day 30. A similar pattern of gene expression changes occurred with 30 days of BMP7 or BMP4 treatment (Figure 2I).

### Omitting or extending BMP7 treatment affects hBA bioenergetics.

**Figure 4:**
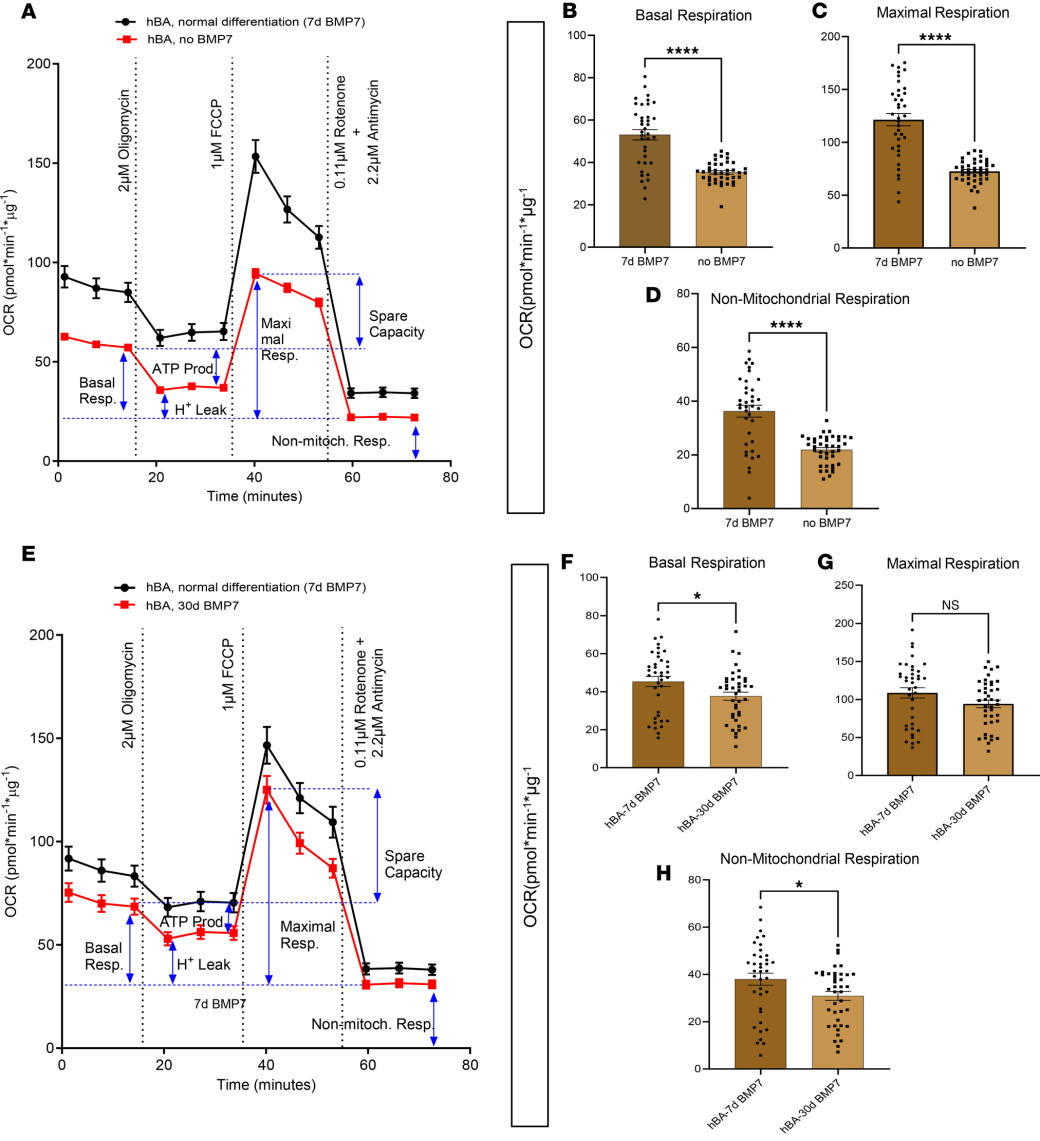
Omitting or extending BMP7 treatment decreased both basal and drug-stimulated cellular bioenergetics in hBAs. Omitting or extending BMP7 treatment decreased both basal and drug-stimulated cellular bioenergetics in hBAs. Mitochondrial stress test with oxygen consumption rate (OCR) tracings in (**A**) hBAs with 7 days of BMP7 or no BMP7 and (**E**) hBAs with 7 days or 30 days of BMP7. Quantified respiratory profile of hBAs showing (**B** and **F**) basal respiration, (**C** and **G**) maximal respiration, and (**D** and **H**) nonmitochondrial respiration (*n* = 35–40 replicates for each treatment group). hBAs treated with the 7-day BMP7 differentiation protocol in black line and light brown bars, and non-BMP7–treated hBAs or 30-day BMP7-treated hBAs in red line and dark brown bars. Comparisons between 7-day BMP7-treated hBAs and non-BMP7–treated or 30-day BMP7-treated hBAs were made using 2-tailed unpaired Student’s *t* test. *P < 0.05; ****P < 0.0001.

Given the time-dependent effects of BMP7 on hBA gene and protein expression, we assessed the influence of BMP7 on hBA cellular bioenergetics. Consistent with our prior transcriptional and protein data, 7 days of BMP7 significantly augmented basal, maximal, and nonmitochondrial respiration compared with cells without BMP7 exposure (Figure 4, A–D). As was reflected in the prior qPCR and immunoblots, 30 days of BMP7 did not further augment the bioenergetic capacity of hBAs but instead led to decreased basal and nonmitochondrial respiration compared with the 7-day BMP7 treatment group (Figure 4, E–H).

### Both BMP7 and BMP4 treatment increased basal respiration and stimulated hWA cellular bioenergetics without detectable UCP1.

**Figure 5:**
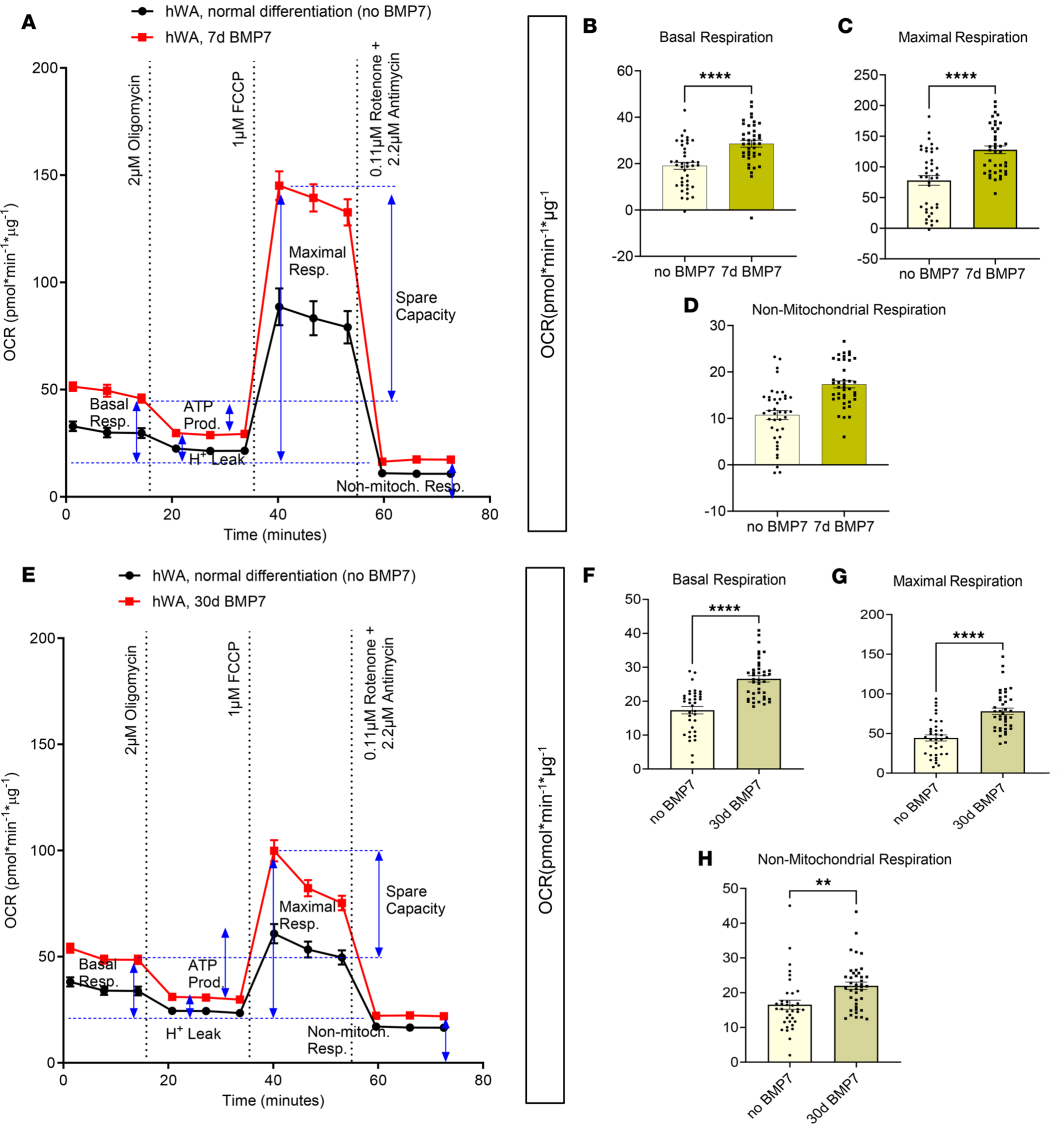
BMP7 treatment increased basal and stimulated cellular bioenergetics in hWAs after 7 or 30 days of BMP7 exposure. BMP7 treatment increased basal and stimulated cellular bioenergetics in hWAs after 7 and 30 days of BMP7 exposure. Mitochondrial stress test with oxygen consumption rate (OCR) tracings in hWAs with (**A**) no BMP7 or 7 days of BMP7 and (**E**) hWAs with no BMP7 or 30 days of BMP7. Quantified respiratory profile of hWAs showing (**B** and **F**) basal respiration, (**C** and **G**) maximal respiration, and (**D** and **H**) nonmitochondrial respiration (*n* = 35–40 replicates for each treatment group). hWAs treated with the standard differentiation protocol in black line and light-yellow bars, and 7-day BMP7-treated hWAs or 30-day BMP7-treated hWAs in red line and dark brown bars. Comparisons between non-BMP7–treated hWAs and 7-day BMP7-treated or 30-day BMP7-treated hWAs were made using 2-tailed unpaired Student’s *t* test. **P < 0.01; ****P < 0.0001.

In the hWAs, the qPCR and Western blot data suggested that BMP7 did not induce transcriptional or protein expression changes expected to affect bioenergetics. Nevertheless, the mitochondrial stress tests demonstrated a general increase in multiple aspects of energy expenditure. Seven-day BMP7 treatment led to higher basal, maximal, and nonmitochondrial respiration relative to hWAs not treated with BMP7 (Figure 5, A–D). Also, 30-day BMP7 treatment resulted in higher basal, maximal, and nonmitochondrial respiration compared with the baseline (Figure 5, E–H). Notably, this increased energy expenditure was produced without expression of the concomitant genetic or protein markers consistent with classically described beige adipocytes, particularly UCP1.

**Figure 6:**
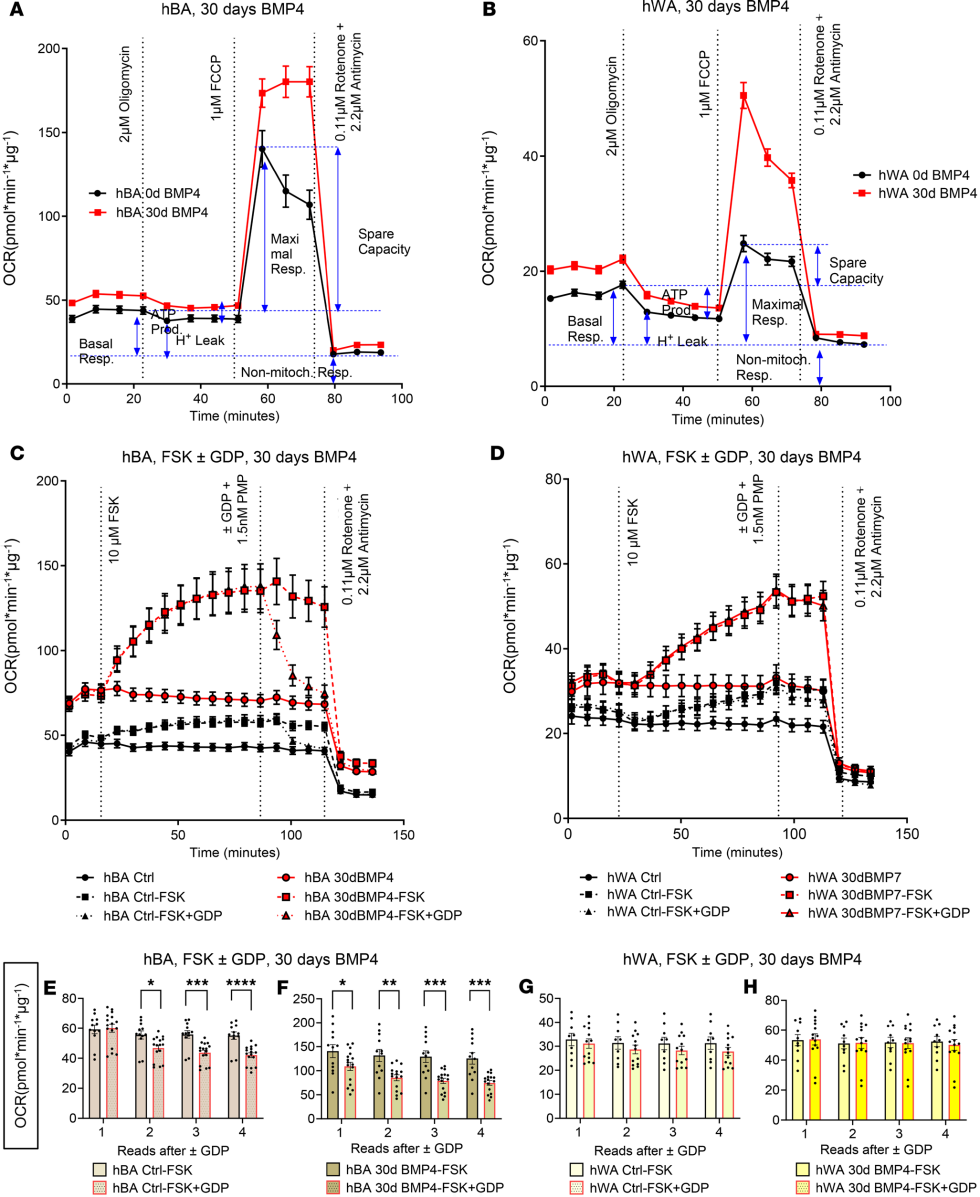
BMP4 treatment for 30 days affected cellular bioenergetics in hBAs and hWAs. BMP4 treatment for 30 days affected cellular bioenergetics in hBAs and hWAs. Mitochondrial stress test (MST) with oxygen consumption rate (OCR) tracings in (**A**) hBAs and (**B**) hWAs with or without FSK and GDP (*n* = 38–40) replicates for each treatment group). hBAs or hWAs treated with 0 days of BMP4 in black line and 30 days of BMP4 in red line. OCR after addition of 10 μM forskolin (FSK) ± GDP in (**C**) hBAs (*n* = 12–16) or (**D**) hWAs (*n* = 10–14) with 30 days of BMP4. Quantification of the 4 OCR reads after GDP treatment in hBAs (**E** and **F**) or hWAs (**G** and **H**). hBA or hWA control in black lines and hBA or hWA with BMP4 in red lines. Comparisons between non-BMP4–treated and 30-day BMP4-treated hBAs and hWAs were made using 2-tailed unpaired Student’s *t* test. *P < 0.05; **P < 0.01; ***P < 0.001; ****P < 0.0001.

Since BMP4 induced similar genetic and protein expression as BMP7 in hBAs and hWAs, we also explored the effects of BMP4 on cellular bioenergetics. Unlike with BMP7, the 7-day BMP4 treatment did not significantly affect any measure of energy expenditure (Supplemental Figure 7, A–D). Rather, it was the full 30-day BMP4 exposure that led to increased basal, maximal, and nonmitochondrial respiration for both hBAs and hWAs (Figure 6, A and B, and Supplemental Figure 8, A–F).

### Thirty-day BMP4 treatment increased basal respiration and stimulated cellular bioenergetics in both hBAs and hWAs, but excess energy expenditure in hWA is not dependent on UCP1 activity.

A defining feature of brown and beige adipocyte physiology is the presence of adrenergically stimulated, UCP1-mediated uncoupled thermogenesis. To determine whether the excess energy expenditure in hBAs and hWAs was a result of UCP1 activation, we treated cells with forskolin (FSK) to stimulate the adrenergic signal transduction pathway. After the cells demonstrated increased oxygen consumption rate (OCR), they were treated with GDP, a purine nucleotide known to inhibit UCP1 activity (22). As expected, hBAs and hWAs treated with BMP4 for 30 days had overall increased basal and nonmitochondrial respiration relative to controls (Supplemental Figure 9, A–D). In hBAs, FSK addition led to an increase in thermogenesis that was reversed after GDP (Figure 6C). In hWAs, FSK also resulted in increased thermogenesis, but there was no change in OCR after addition of GDP (Figure 6D). hBAs with 30 days of BMP4 treatment showed an accelerated decrease in OCR after addition of GDP relative to the hBA control group (Figure 6, E and F). In contrast, both controls and hWAs treated with 30 days of BMP4 were unaffected by addition of GDP (Figure 6, G and H). These results indicate that the excess energy expenditure seen in BMP4-treated hWAs cannot be attributed to UCP1.

### RNA-Seq analysis identifies Gene Ontology pathways that were different in BMP7-treated hWAs.

RNA-Seq showed that certain Gene Ontology processes were enriched in hWAs treated for 7 days with BMP7. The pathways that could be associated with the increased basal, maximal, and nonmitochondrial respiration shown in Figure 5 include fatty acid beta-oxidation, positive regulation of cholesterol transport, mitochondrial membrane organization, and cellular glucose homeostasis (Supplemental Figure 10, A and B). We also used RNA-Seq to compare the gene expression profile of the BMP-treated and standard hWAs with structural Wnt-regulated adipose tissue-resident (SWAT) cells. Human brown and white adipose depots separate into 2 main cell fates, an adipogenic and a structural branch, developing from a common progenitor (23). SWAT cells are progenitor cells found in human WAT and BAT depots that have a structural rather than an adipogenic fate. Deconvolution of the RNA-Seq data using CIBERSORTx and a gene signature set that included SWAT, adipogenic, and progenitor cell types (23) showed that the predicted abundance was highest for adipogenic cells (~80%) followed by progenitor cells (~20%) with a noticeable absence of SWAT cells except for a minute fraction (1.5% to 3.5%) in 2 out of the 6 hWA datasets tested (Supplemental Figure 11, A and B). Therefore, we cannot exclude the possibility that the hWA cells have a functional relationship with SWAT cells. In contrast, deconvolution showed absence of SWAT cell signatures in all datasets of hWA cells treated with BMP7. Further studies are necessary to explore these 2 cell types in greater detail.

### The higher energy expenditure in BMP7-treated hWAs is substantially accounted for by futile creatine cycling.

Besides UCP1-mediated mitochondrial uncoupling, other processes have been shown to increase thermogenesis in adipocytes. Recent studies have pointed to futile creatine cycling as a relevant mechanism in both UCP1-positive and UCP1-negative mouse cells (24–26). A key enzyme mediating this process is tissue nonspecific alkaline phosphatase (TNAP), which can be inhibited by 2,5-dimethoxy-N-(quinolin-3-yl) benzene sulfonamide (DQB) (27). hWAs differentiated either without or with 7 days of BMP7 were incubated for 30 minutes with DQB over a range of concentrations and then treated with 10 μM FSK to increase OCR. At the highest concentration, 100 μM, DQB inhibited 80% of OCR in BMP7-treated hWAs (*P* = 1.2 × 10^–8^), but there was no significant effect on OCR in hWAs (Supplemental Figure 12, A–D). These data indicate that a substantial proportion of OCR in BMP7-treated hWAs — though not all — was due to the thermogenic futile creatine cycle.

## Discussion

Human adipose tissue exists along a functional spectrum with energy-storing white adipocytes and energy-expending brown adipocytes situated on either end. Beige adipocytes lie in the middle, and their existence has prompted interest in factors that can induce WAT to be more metabolically active and energy expending. In this study, we successfully used the TGF-β signaling pathway to increase mitochondrial respiration in human white adipocytes by treatment during differentiation with either BMP7 or BMP4. Of note, this increased energy expenditure in differentiated hWAs was not accompanied by increased expression of UCP1 and was not blunted by addition of the UCP1 inhibitor GDP. Rather, the majority of the uncoupled thermogenesis came from futile creatine cycling. This phenotype, with higher energy expenditure but no UCP1, is unlike the typical beige adipocyte, which is defined by expression of UCP1 (28). We propose that these cells may represent a distinct class of white adipocytes with UCP1-independent increases in energy expenditure, in between white and beige. These distinct cells are relevant to the prospect of “beiging” white adipocytes in a WAT depot as a therapeutic strategy to treat cardiometabolic diseases, which has drawn substantial interest. It remains unclear whether beige adipocytes can be safely and reliably induced in human WAT, particularly the large subcutaneous depots, and by what mechanism (6). Our characterization of these adipocytes offers another possible route: induction of heightened energy expenditure in white adipocytes can occur even without bona fide differentiation into beige adipocytes. In aggregate, similar activation of a portion of the total volume of WAT may provide substantial metabolic benefits.

Beyond the effects on energy expenditure and adipocyte-specific genetic markers, we were able to investigate how the effects of BMP7 and BMP4 may be mediated through the TGF-β signaling pathway. There was a dynamic trend across 30 days of differentiation. The general decline in TGF-β–responsive genes over the first 14 days may serve to maintain a blockade on proliferation during early phases of differentiation, allowing more commitment of cellular resources toward production of proteins necessary for thermogenesis. Additionally, several studies have demonstrated that activation of the TGF-β signaling pathway inhibits adipogenesis and maturation of brown adipocytes (29, 30). Inhibition of TGF-β/Smad3 signaling in mice is associated with protection from obesity and diabetes and leads to WAT acquiring a bioenergetic and genetic profile more similar to brown fat/skeletal muscle (31). In addition, GPR180, a member of the TGF-β signaling cascade, has been found to mediate Smad3 phosphorylation and promote thermogenic activity in mature brown and beige adipocytes (32), further linking the TGF-β pathway to adipocyte thermogenic function. As such, beyond inhibiting proliferation, repression of TGF-β signals during differentiation may serve the dual purpose of facilitating differentiation to a more energy-expending phenotype.

We were also able to explore nuances in the conditions required for BMP7 and BMP4 to institute their previously described thermogenesis-promoting effects on hBAs. Although 7-day BMP treatment increased the thermogenic function of hBAs, a full 30-day treatment decreased hBA-defining genes and impaired mitochondrial function. Previous studies have not clarified the effects of chronic BMP exposure on immortalized human adipocytes. Our results may reflect the physiological observation that chronic exposure to TGF-β signaling leads to dysfunctional adipose tissue (31, 33). The role of BMP4 in adipogenesis has been poorly defined. In one model, BMP4 led to a white lineage (9); others found that treatment helped commit to a brown lineage (11, 34); yet in a different model, BMP4 drove differentiation to white and brown (35). A previous study of human white adipose stem cells stimulated with BMP4 and BMP7 found that both BMPs induce a transition to a beige-like transcriptional profile, with increased expression of *UCP1* and decreased expression of the white adipocyte marker *TCF21* (11). Of note, the study noted that there was great donor-dependent variability in the response to BMP4 or BMP7 (11). In our present study, we report a similar reduction in *TCF21* expression in response to BMP4 or BMP7 treatment. However, we found that *UCP1* expression was unaffected by BMP4 or BMP7 in hWAs. Altogether, our contrasting results on the effects of BMP treatment on *UCP1* expression reflect underlying functional heterogeneity in human adipocytes. In terms of translation to human metabolism, recombinant human BMP2 and BMP7 have been used for bone regeneration (36). In addition, there is evidence that BMPs can affect rodent glucose metabolism (37–39). If BMP-based energetic activation of white preadipocytes is to become a viable therapeutic strategy, further work is required to understand what factors contribute to sensitizing adipocytes to BMPs and what the optimal BMP exposure is to facilitate improved energy expenditure in hBAs and hWAs.

A limitation of this study is that these phenomena were seen in 2 cell lines, hWAs from the abdominal subcutaneous depot and hBAs from the perirenal depot, originating from a 47-year-old male. Although we have conducted extensive characterization of these cell lines to ensure they behave in a physiologically representative manner, our results may not capture variations driven by adipose tissue depot location and type of mammalian adipose tissue cell line (13). Human adipose tissue is known to be heterogenous, exhibiting inter-depot and intersubject differences. A prior study reported variations in BAT gene expression and responses to stimuli across hBAs derived from different human subjects (12). Even within our present study, microarray data from hBAT-SVF cells derived from the deep neck showed an increase in white adipocyte marker *HOXC8*, which contrasts with the pattern seen in our own hBA model. Bearing in mind that our model systems may not recapitulate the full diversity of human adipose tissue, some generalizability of our findings is supported by studies in mice that have similarly described subpopulations of *UCP1*-negative beige adipocytes or low-*UCP1* brown adipocytes (25, 40). Our findings suggest that similar subpopulations may exist in human adipose tissue and demonstrate that energy expenditure within this subpopulation of human adipocytes is inducible through BMP treatment. In addition, although we report increased energy expenditure in our adipocyte subpopulation that it is attributable to the futile creatine cycle and upregulation of catabolic pathways, the precise mechanisms and physiological settings remain to be determined (24).

In summary, we found that members of the TGF-β protein family, BMP7 and BMP4, push hBAs and hWAs toward a more energy-expending phenotype. Our findings provide an opportunity to further explore the conditions and mechanisms by which bioenergetic capacity of adipocytes is regulated and provide insight into how to capitalize on the metabolic plasticity of human adipocytes to develop therapies for obesity and related diseases.

## Methods

### Differentiation of immortalized and clonal hBAs and hWAs.

Immortalized, clonal, hBAs and hWAs were generated as described by us and ATCC (13). Cells were maintained in growth media containing DMEM/F12 medium (Gibco) supplemented with 10% FBS (ATCC) and 1% penicillin-streptomycin until 100% confluency was reached. Differentiation began 48 hours afterward.

hBAs and hWAs underwent 1 of 3 differentiation protocols: protocol 1 (control), protocol 2 (7-day BMP7 exposure), and protocol 3 (30-day BMP7 exposure). Descriptions of differentiation media 1–4 are provided in Supplemental Table 2.

Protocol 1 (control) followed the protocol for hWAs as described previously (13). Differentiation took place over 30 days using 2 related media: for the first 7 days, cells were incubated in differentiation media 1, containing DMEM/F12 medium, 2% FBS, 1% penicillin-streptomycin, 0.1 μM dexamethasone, 0.5 mM 3-isobutyl-1-methylxanthine (IBMX), 33 μM biotin, 2 nM triiodothyronine (T3), 30 μM indomethacin, 17 μM pantothenate, and 2 μM rosiglitazone. For the remaining 23 days, cells were incubated in differentiation media 2, which had the same content as differentiation media 1 except rosiglitazone was not included. For protocol 2 (7-day BMP7 exposure), preadipocytes were exposed to 7 days of differentiation media 3, which had the same components as differentiation media 1 (above), in addition to 3.3 nM BMP7 or BMP4 (both R&D Systems). Subsequently, for the remaining 23 days, the cells were exposed to differentiation media 2. For protocol 3 (30-day BMP7 exposure), cells were exposed to 7 days of differentiation media 3. For the remaining 23 days of the differentiation process, group 3 was exposed to differentiation media 4, which contained the same reagents as differentiation media 2 but with the addition of 3.3 nM BMP7 or BMP4.

### mRNA expression in hBAs and hWAs.

mRNA from hWAs and hBAs was isolated as described (9). In brief, total RNA was extracted from cells grown on 6-well plates using 500 μL TRIzol reagent (Invitrogen, Thermo Fisher Scientific) per well following the manufacturer’s protocol. RNA quantity and quality were determined by spectrophotometry (NanoDrop, Thermo Fisher Scientific). A total of 1 μg of RNA was reverse-transcribed using High-Capacity cDNA Reverse Transcription kit (Applied Biosystems, Thermo Fisher Scientific). Quantitative reverse transcription PCR (qPCR) was run in duplicates using SYBR green fluorescent dye (Bio Basic) and quantified in the QuantStudio 6 Flex Real-Time PCR System (Applied Biosystems, Thermo Fisher Scientific) using the standard SYBR green thermal cycling conditions. Each point on a PCR graph represents a biological replicate and is the average of 2 technical replicates. All statistical analyses were based solely on biological replicates. The number of independent data points in a graph was determined by 2 factors. The first was primer efficiency among genes. Individual points were excluded when (a) there was not a well-defined, “clean” melting curve that would indicate efficient specific primer amplification and (b) the Ct values between technical replicates showed too much variation. Second, across the 30-day differentiation period, some wells’ cells did not grow and progress to the final stage for assessment. Relative mRNA expression was determined by the Δ-Ct method using actin as an endogenous housekeeping control. All sequences of primers used in this study are provided in Supplemental Table 1. When comparing gene expression between different experimental variables in hBAs and hWAs, gene expression was normalized to protocol 1, or the control.

### Western blot analysis.

Western blotting was done using preadipocytes or differentiated adipocytes as described (9). In brief, cells were washed twice with ice-cold PBS and lysed in RIPA buffer (50 mM Tris pH 7.4; 150 mM NaCl; 1 mM EDTA; 1% Triton X-100; 0.1% SDS) containing cOmplete Protease Inhibitor Cocktail (Roche). Cell lysates were sonicated and clarified by centrifugation at 14,000*g* for 20 minutes at 4°C. Protein concentrations of cell supernatants were determined using the bicinchoninic acid reagents (Pierce, Thermo Fisher Scientific) using BSA as the standard. Proteins (20 μg) were separated by SDS-PAGE (Bio-Rad) (4% to 20% gradient gels) and transferred to PVDF membranes (Bio-Rad). Membranes were blocked in Tris-buffered saline (pH 7.5) containing 0.05% Tween 20 and 5% blotting-grade blocker (Bio-Rad) for 1 hour at room temperature and then probed with primary antibodies: anti-β-actin mouse mAb (A2228, MilliporeSigma, 1:5,000) and anti-UCP1 antibody produced in rabbit (ab155117, Abcam, 1:500), along with OxPhos Rodent WB Antibody Cocktail (45-8099, Invitrogen, Thermo Fisher Scientific, 1:1,000), PPARγ (PA3-821A, Invitrogen, 1:1,000), and PGC1α (ab188102 Abcam 1:250) overnight at 4°C. Membranes were washed with Tris-buffered saline (pH 7.5), and then probed with secondary antibodies: mouse (7076P2, Cell Signaling Technology) or rabbit (7074P2, Cell Signaling Technology) for 1 hour at room temperature. Visualization of bands was done using enhanced chemiluminescence.

### Oil Red O staining.

As described previously (9), cells were washed twice with PBS, fixed with 4% paraformaldehyde for 1 hour, and stained with filtered Oil Red O solution (0.5% Oil Red O in isopropyl alcohol) for 4 hours at room temperature. Cells were then washed with PBS, and a photograph of the dish was taken.

### RNA-Seq and analysis.

hWA cells were cultured as described earlier. In 10 cm plates, cells were seeded at 1.5 × 10^6^ cells per plate and allowed to adhere overnight before experimental treatments. Cells were differentiated as described earlier either in the presence (hWA-BMP7) or absence (hWA-control) of BMP7. Six replicates were prepared for each condition. At the experimental endpoint, cells were washed twice with ice-cold PBS and lysed directly in TRIzol reagent (Invitrogen). Total RNA was extracted using Zymo Direct-zol RNA Miniprep kit (Zymo Research) according to the manufacturer’s protocol, and RNA quality was assessed using an Agilent TapeStation (Agilent Technologies). RNA preparations with RNA Integrity Number of 9.0 or higher were further processed for library preparation. Strand-specific RNA-Seq libraries were prepared using the VAHTS Universal V10 RNA-Seq Library Prep kit (Illumina) following the manufacturer’s instructions. Libraries were quantified by Qubit fluorometric quantitation (Thermo Fisher Scientific) and quality-checked by qPCR. Pooled libraries were sequenced on an Element Biosciences AVITI platform with a 2 × 150 cycle sequencing kit using paired-end 150 bp reads, targeting 60–80 million read pairs per sample.

Raw sequencing reads were processed using a customized RNA-Seq pipeline based on ARMOR (41), a Snakemake-based workflow for comprehensive RNA-Seq analysis. Quality control of raw reads was performed with FastQC (v0.12.1) and MultiQC (v1.28). Adapter sequences and low-quality bases were trimmed using TrimGalore (v0.6.7). Processed reads were aligned to the human reference genome (GRCh38/hg38) using STAR aligner (v2.7.11b) with default parameters. Further, bedtools (v2.31.1) and samtools (1.21) were used for processing. In parallel, transcript-level quantification was performed using Salmon (v1.10.1) with decoy-aware indexing against the GENCODE v49 transcriptome to account for potential alignment artifacts. Gene-level counts were aggregated from transcript abundances using tximport (v1.22.0).

Differential gene expression analysis was conducted using pyDESeq2 (v0.5.2) in Python (v3.10.0) or DESeq2 (v1.34.0) in R (v4.4) on STAR-derived and Salmon-derived count matrices, respectively. Genes with fewer than 10 counts across all samples prior to analysis and genes with baseMean values less than 10 after analysis were filtered out. Differentially expressed genes were identified using a Wald test with Benjamini-Hochberg adjusted *P* value less than 0.05.

Gene set enrichment analysis (GSEA) was performed using GSEA.py (v1.1.10) or GSEA 4.4.0 (R) with gene sets from the Molecular Signatures Database (MSigDB v2023.1.Hs), including Gene Ontology Biological Process collections. Pre-ranked GSEA was conducted using log_2_ fold-changes from DESeq2 output as the ranking metric. Gene sets with FDR *q* value less than 0.25 were considered significantly enriched. Results were visualized using bubble plots in Python or R.

### Bulk RNA-Seq deconvolution.

We applied CIBERSORTx-based deconvolution (42) to identify overlapping single-cell RNA-Seq–based gene signatures of SWAT cells, progenitor cells, and adipogenic cells with those present but not detected in bulk RNA-Seq hWA and hWA-BMP7 datasets. This approach allowed us to estimate the abundance of the mentioned cell types from bulk cell transcriptomics. For mixture files and bulk RNA-Seq data, gene-level counts that had been aggregated from transcript abundances (Salmon) using tximport were used. For signature matrix files and single-cell RNA-Seq data, we used a gene signature set that included SWAT, adipogenic, and progenitor cell types (23). The following run conditions were applied: batch mode “B,” quantile normalization “off,” run mode “relative,” and number of permutations “100.” All analyzed samples had *P* values less than 0.0001, root mean square error less than 1, and Pearson’s coefficients (observed vs. predicted) of 0.9 or higher. A Wilcoxon rank-sum test was performed for statistical testing. No statistical differences were observed between hWA and hWA-BMP7 bulk RNA-Seq data in relationship to SWAT, adipogenic, or progenitor cell-type signatures.

### Lipid droplet visualization and image processing, cell culture, staining, and image acquisition.

Cells were cultured as described earlier. Cells were seeded at 40,000 cells/well in 8-well chamber slides (Corning) and transdifferentiated for 30 days with or without BMP7 as described earlier. For live-cell staining, media was replaced with fresh media, and cells were incubated with Vybrant DyeCycle violet (5 μM, Invitrogen) for nuclear labeling and CellTracker deep red (1 μM, Invitrogen) for cytoplasmic labeling at 37°C for 30 minutes. After staining, cells were fixed with 4% paraformaldehyde (PFA) in PBS for 15 minutes at room temperature. After fixation, neutral lipids were labeled with BODIPY 493/503 (2 μg/mL, Invitrogen) for 20 minutes. Wells were washed 3 times with PBS, mounted with ProLong Gold Antifade Mountant (Invitrogen), and coverslipped.

Fluorescence imaging was performed on an Olympus VS200 slide scanning system (Evident Corporation) using a 20× objective (numerical aperture 0.75), yielding a pixel resolution of 0.3251 μm/pixel. Image fields of 4,096 × 4,096 pixels were acquired in 3 channels: DAPI (excitation 405 nm) for Vybrant DyeCycle violet, FITC (excitation 488 nm) for Bodipy 493/503, and Cy5 (excitation 640 nm) for CellTracker deep red. Images were saved in VSI format with associated metadata.

### Image processing and segmentation.

Image files were imported into QuPath (v0.4.3) for initial processing and quality control. Individual image fields were subdivided into 9 fields to facilitate downstream analysis. Regions of interest (ROIs) of 1,024 × 1,024 pixels were extracted from each field using Fiji (ImageJ, NIH, v1.53c). Image preprocessing was performed using BioVoxxel Toolbox and standard Fiji plugins, including pseudo-field correction (blurring = 50) and Enhance Local Contrast (CLAHE) (blocksize = 60, histogram = 256, maximum = 2, mask = *None) to optimize signal-to-noise ratios.

For each experimental group (hWA control and hWA-BMP7), 32 representative ROIs were selected for quantitative analysis. Nuclear and lipid droplet segmentation was performed using Cellpose (v4.0.8), a deep learning–based segmentation algorithm. For nuclear segmentation, the nuclei pretrained model was applied with a diameter parameter of 45.9 pixels and a standard flow threshold. Lipid droplet segmentation utilized the cyto3 model with a diameter of 20.5 pixels and standard flow threshold. Segmentation masks were exported as labeled 16-bit TIFF images for downstream quantification. Total nuclei (hWA): 5,295; (hWA_BMP7): 5,311.

### Quantitative analysis and statistics.

Segmentation masks for nuclei and lipid droplets were analyzed using custom Python scripts (Python v3.10) incorporating scikit-image (v0.25.2), scipy (v1.16.3), NumPy (v2.3.5), and pandas (v2.3.3) libraries.

For each segmented ROI, lipid droplets were classified based on local spatial density and neighborhood properties, with multilocular lipid droplets associating with a high density of small lipid droplets and unilocular lipid droplets associating with isolated large lipid droplets or low-density regions. Lipid droplet classes, unilocular or multilocular, were computationally assigned to individual features on respective segmentation maps. Based on these assignments, metrics including nuclei and lipid droplet count and their area fraction, lipid droplet size and density distribution, and class frequencies were determined.

All statistical analyses were performed in Python using SciPy (v1.16.3) and statsmodels (v0.14.6). Normality of distributions was assessed using the Shapiro-Wilk test. For normally distributed data, differences between hWA and hWA-BMP7 groups were evaluated using 2-tailed Welch’s *t* test. Non-normally distributed data were analyzed using the Mann-Whitney *U* test. Data visualization was performed using Matplotlib (v3.10.8) and seaborn (v0.13.2).

### Cellular bioenergetics assays.

Cellular OCRs were measured as described (9). Preadipocytes grown to 90% confluence from a 10 cm cell culture dish were plated into individual wells of XF96 cell culture microplates (Agilent Cell Analysis Technology). Once confluent, the population of cells in each were differentiated into adipocytes as described above. After differentiation and transfection, real-time OCRs were assessed using the Seahorse XFe Extracellular Flux Analyzer. For the mitochondrial stress assay, on the day of the experiment, primary adipocytes were washed in prewarmed XF assay media supplemented with sodium pyruvate (1 mM), l-glutamine (2 mM), and glucose (25 mM) and adjusted to pH 7.4. Cells were then maintained in the same assay buffer in a non-CO_2_ incubator for 1 hour. Cellular respiration was analyzed by using the following perturbation drugs: 2 μM oligomycin, 2 μM FCCP, and respiratory chain inhibitors 0.11 μM rotenone and 2.2 μM antimycin A. Cellular respiration was also measured for select groups of cells after treatment with 10 μM FSK, 1.5 nM XF plasma membrane permeabilizer, and 3 mM GDP.

All drugs were loaded together into the injection ports in the XFe-96 sensor cartridge, and the XF96 analyzer was operated under the manufacturer’s basal protocol at 37°C. Measurements were normalized by protein content (BSA assay). Nonmitochondrial respiration was subtracted from basal, uncoupled, and FCCP respiration, a standard calculation adjustment that is part of the Seahorse XF Cell Mito Stress Test Report Generator. Data were then normalized to basal OCR of each transfection treatment. The dataset was analyzed by XFe-96 software and GraphPad Prism, and energy plots were generated by following the manufacturer’s guidelines and instructions (Seahorse/Agilent).

### Microarray.

Isolation of cellular material and performance of the microarray were done as described previously (12). Analysis of gene expression using GeneChip PrimeView (Affymetrix) was performed on highly adipogenic clonal brown cell lines either treated with BMP7 or untreated. RNA was isolated from clonal cell lines using Direct-zol RNA MiniPrep kit (Zymo Research) according to the manufacturer’s instructions. Biotin-labeled cRNA was synthesized, purified, and fragmented using GeneChip 3′ IVT Express kit (Affymetrix). Integrity and fragmented cRNA were assessed by running aliquots on the Agilent 2100 Bioanalyzer before proceeding further. The high-quality cRNA met the following criteria: the A260/A280 was within values of 1.9–2.0, the 28S/18S rRNA bands (from the gel) were crisp, and the intensity of the 28S band was roughly twice the intensity of the 18S band. Array hybridization and scanning were performed by the Advanced Genomics and Genetics Core of Joslin Diabetes Center according to established methods. Microarray data were normalized using robust multiarray average, which placed it on a log_2_ scale. Normalized data for each probe set were correlated to log_2_(UCP1) using Pearson’s correlation with a t2-sided alternative (with R function cor.test), which yielded correlation coefficients and *P* values. *P* values were adjusted for multiple testing using FDR with the p.adjust R function.

### TNAP inhibition study.

hWA cells were cultured in DMEM, seeded at 10,000 cells/well on Seahorse XFe96/XF Pro 96-well plates (Agilent), and then transdifferentiated as described earlier either in the presence (hWA-BMP7) or absence (hWA-control) of BMP7. TNAP inhibitor DQB (613810, Millipore Sigma) was dissolved in DMSO and diluted to desired concentrations in Seahorse XF DMEM media (pH 7.4) supplemented with glucose, glutamate, and pyruvate per the manufacturer’s protocol.

On the day of the assay, culture medium was replaced with supplemented Seahorse XF DMEM media and plates were incubated at 37°C in a humidified chamber (room air). Media was replaced with DQB-containing media 30 minutes prior to the Seahorse assay (XFe96 Analyzer) and incubation continued. To minimize inhibitor washout, the media replacement was omitted prior to baseline OCR measurement. FSK was injected to a final concentration of 10 μM after 3 measurement cycles (mix 3 minutes, wait 0 minutes, measure 3 minutes) and OCR was recorded over 48 minutes, after which rotenone/antimycin A (0.11 μM/2.2 μM, respectively) were co-injected and recording continued. Data were exported using Seahorse Wave Controller Software (v2.6.4.24) and analyzed using Seahorse Analytics (https://seahorseanalytics.agilent.com/) and custom Python scripts.

### Statistics.

Data are presented as mean ± SEM. Significance between groups was determined using a 2-tailed unpaired Student’s *t* test or 1-way or 2-way ANOVA when appropriate with multiple comparisons (9). Calculations were performed using GraphPad Prism software version 10. For statistical testing, Shapiro-Wilk, Welch’s *t* test, and Mann-Whitney *U* test were applied, and *P* values were adjusted for multiple testing using the Benjamini-Hochberg correction. Statistical significance was defined as *P* less than 0.05.

### Study approval.

This study followed the institutional guidelines of and was approved by the Human Studies Institutional Review Boards of Beth Israel Deaconess Medical Center and Joslin Diabetes Center in Boston, MA. All subjects gave written informed consent prior to participation.

### Data availability.

Data are provided in the Supplemental Data Values file associated with this manuscript.

## Author contributions

KTL, CC, AMC, JH, and SGR designed the experiments. KTL, JH, and AMC wrote the manuscript. KTL, CC, SLA, NN, JHK, JH, and ARG prepared primary brown/beige adipocytes and performed all the experiments. KTL, CC, SLA, NN, JHK, ARG, JH, YHT, SGR, JMD, and YJB analyzed data. KTL, CC, SLA, NN, JHK, ARG, and JH provided technical assistance with experiments on the immortalized adipocytes. KTL, JH, and AMC reviewed and edited the manuscript with input from all authors. CC and AMC supervised the project.

## Conflict of interest 

The authors have declared that no conflict of interest exists.

## Funding support

This work is the result of NIH funding, in whole or in part, and is subject to the NIH Public Access Policy. Through acceptance of this federal funding, the NIH has been given a right to make the work publicly available in PubMed Central.

- Intramural Research Program of the NIDDK within the NIH (DK-075112, DK-075115, and DK-075116, to AMC).
- US National Institutes of Health (NIH) grants R01DK077097 (to YHT), R01DK099511 (to LJG), K23DK081604 (to AMC) and P30DK036836 (to Joslin Diabetes Center’s Diabetes Research Center, DRC) from the National Institute of Diabetes and Digestive and Kidney Diseases.
- Sponsored research grant from Chugai Pharmaceutical Co., Ltd (to YHT and AMC).
- Research grant from the American Diabetes Association (ADA 7-12-BS-191, to YHT).
- Funding from the Harvard Stem Cell Institute (to YHT).

## Supplementary Material

Supplemental data

Unedited blot and gel images

Supplemental table 1

Supplemental table 2

Supporting data values

## Version 1 03/12/2026

In-Press Preview

## Version 2 04/22/2026

Electronic publication
