# Supplementary material for: Bone morphogenetic proteins 4 and 7 increase human white and brown adipocyte thermogenic capacity
Source: JCI Insight. 2026 Mar 12;11(8):e194140. doi: 10.1172/jci.insight.194140 (PMC13135406; doi:10.1172/jci.insight.194140)
Supplement: Supplemental data [file jciinsight-11-194140-s127.pdf]

Supplemental Figure 1

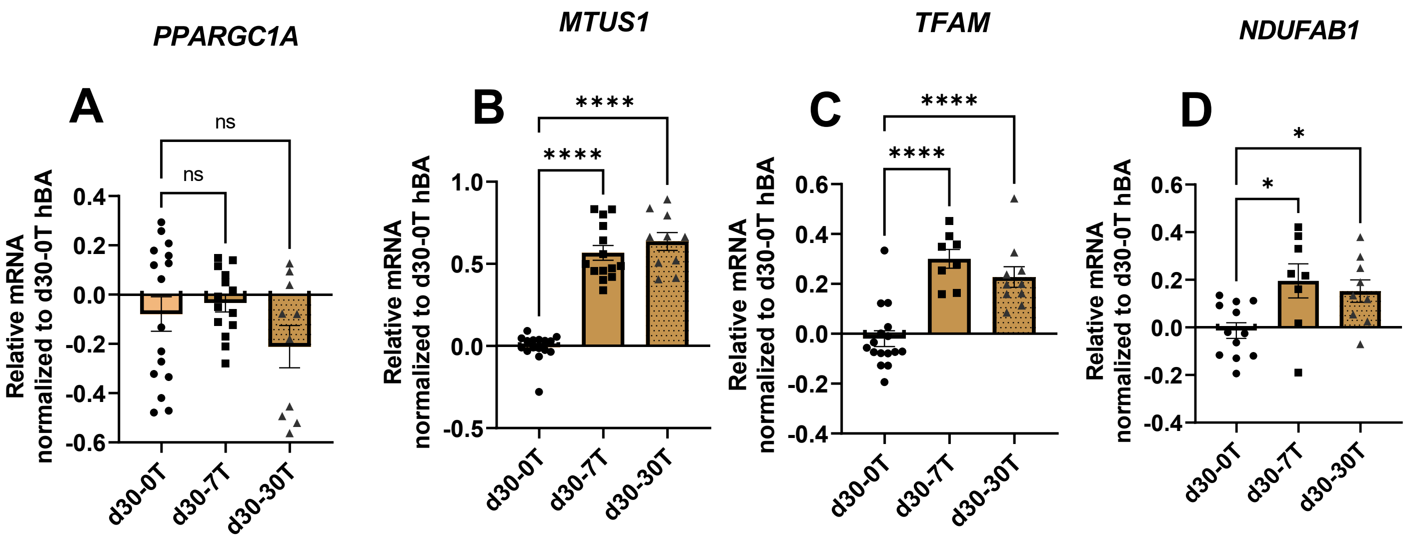

Supplemental Fig. 1. qPCR analysis of hBA adipocytes at 30 days of differentiation without BMP7 treatment (light brown bars), 30 days of differentiation with 7 days of BMP7 treatment (dark brown bars), and 30 days of differentiation with 30 days of BMP7 treatment (checkered dark brown bars). The mRNA expression profiles of **(A)** thermogenic markers, **(B)** brown adipocyte markers, **(C)** mitochondrial markers, and **(D)** electron transport chain markers, *P < 0.05; **P < 0.01; ***P < 0.001; ****P < 0.0001. (n=8-16 replicates). Each data point shown represents a biological replicate.

Supplemental Figure 2

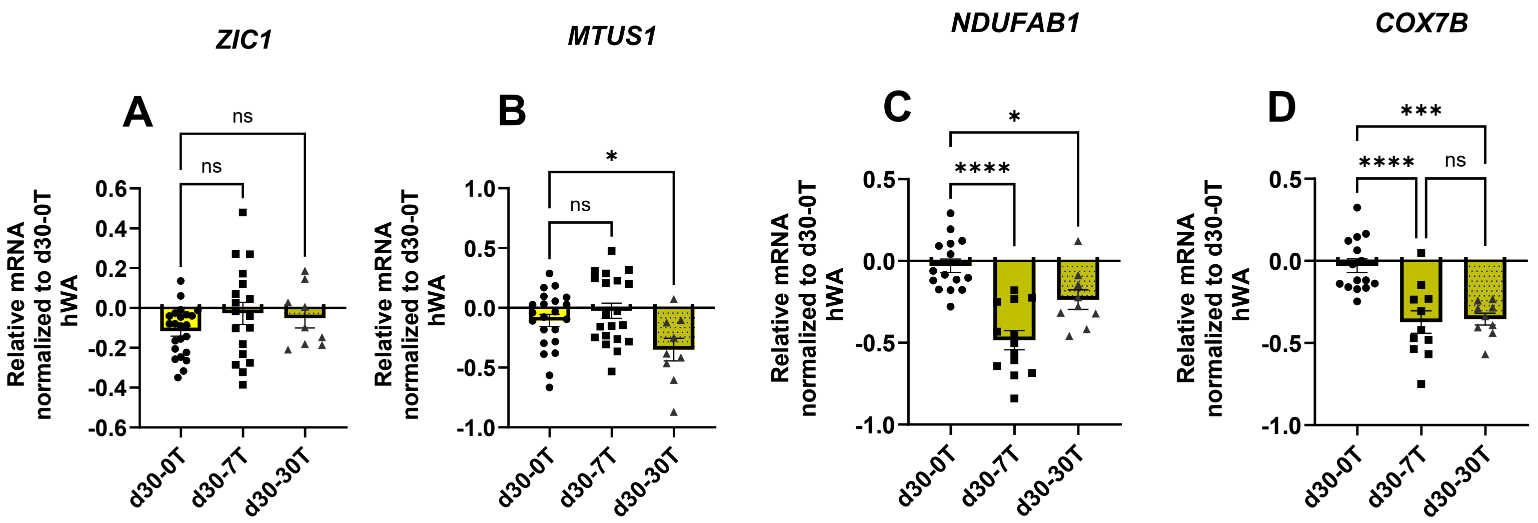

Supplemental Fig. 2. qPCR analysis of hWA adipocytes at 30 days of differentiation without BMP7 treatment (yellow bars), 30 days of differentiation with 7 days of BMP7 treatment (dark yellow bars), and 30 days of differentiation with 30 days of BMP7 treatment (checkered dark yellow bars). The mRNA expression profiles of **(A-B)** brown adipocyte markers and **(C-D)** electron transport chain markers, *$P < 0.05$; **$P < 0.01$; ***$P < 0.001$; ****$P < 0.0001$ (n=8-16 replicates). Each data point shown represents a biological replicate.

Supplemental Figure 3

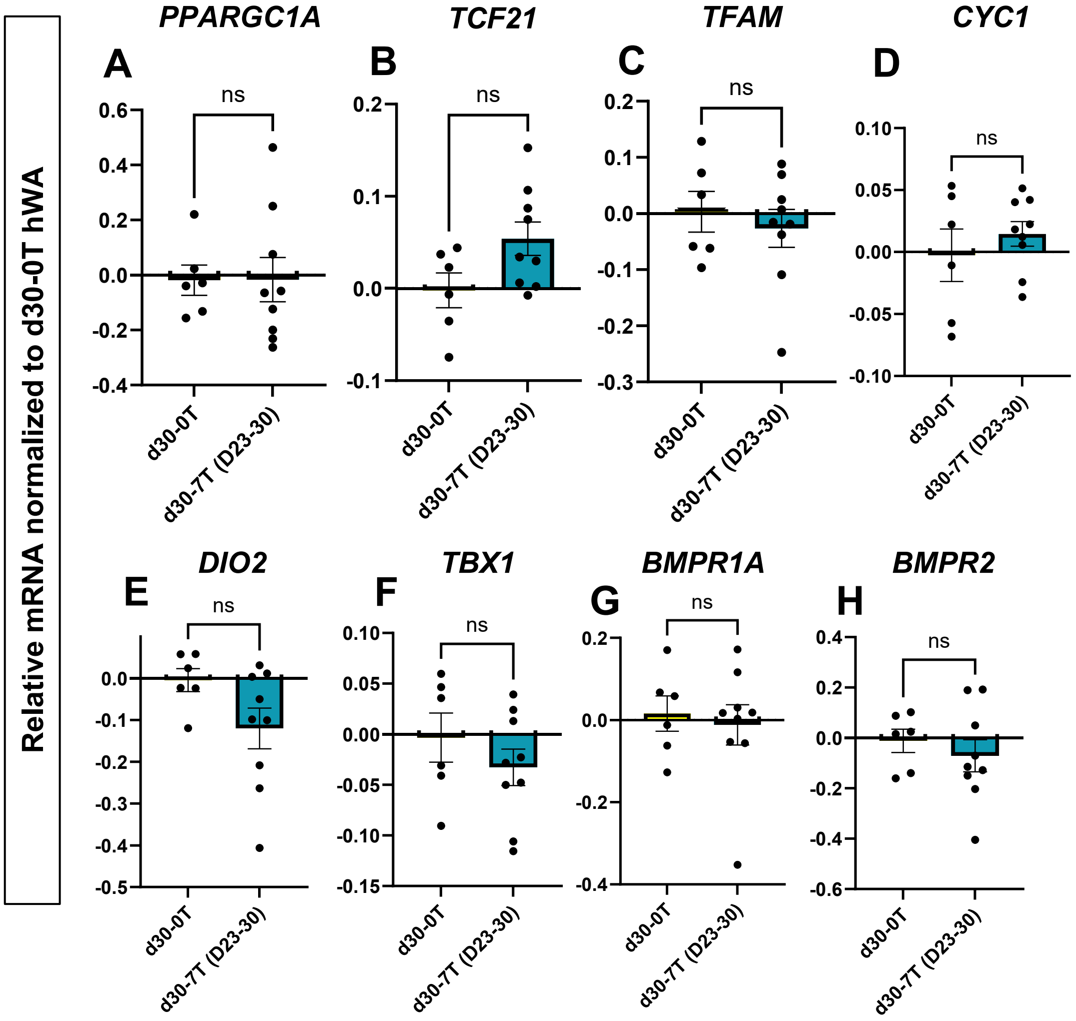

Supplemental Figure 3. qPCR analysis of hWA adipocytes at 30 days of differentiation without BMP7 treatment (yellow bars), 30 days of differentiation with BMP7 treatment at D23 – 30 (blue bars). The mRNA expression profiles of **(A)** thermogenic marker, **(B)** white adipocyte marker, **(C)** mitochondrial marker, **(D)** electron transport chain marker, **(E)** brown adipocyte marker, **(F)** beige adipocyte marker**,** and **(G, H)** BMP receptors. *ns* $P \geq 0.05$; * $P < 0.05$; ** $P < 0.01$; *** $P < 0.001$; (n=6-9 replicates); each data point shown represents a biological replicate.

Supplemental Figure 4

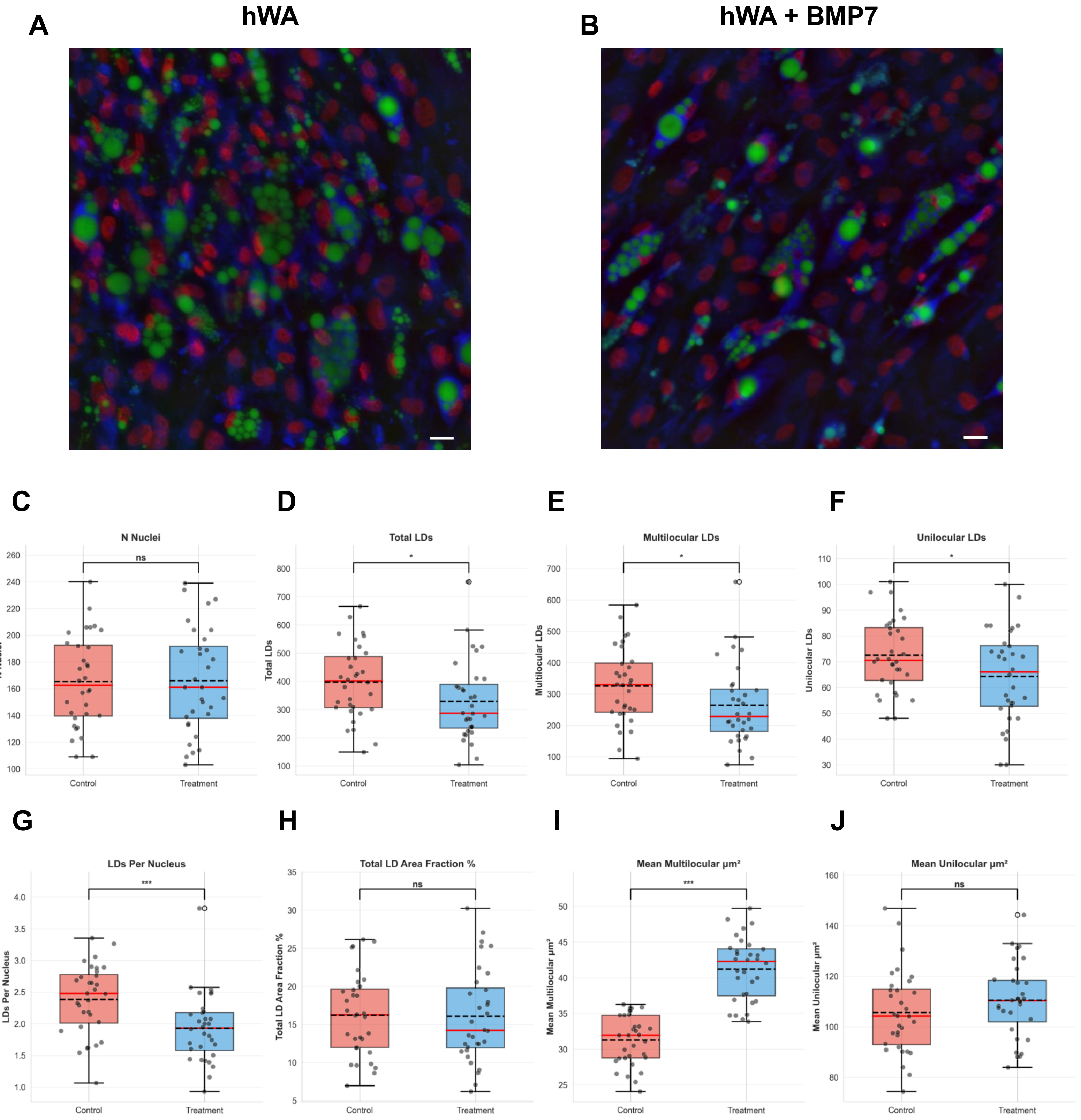

Supplemental Fig. 4. Fluorescence images of hWA differentiated (**A**) without or (**B**) with BMP7 (d0-d7). Red are nuclei; green are lipid droplets, and blue is cytoplasm. Scale bar: 20 µm. Comparison of hWA differentiated without (red) or with BMP7 (d0-d7), "tan cells" (blue). (**C**) Number of nuclei, (**D**) total lipid droplets, (**E**) multilocular lipid droplets, (**F**) unilocular lipid droplets, (**G**) lipid droplets per nucleus, (**H**) total lipid droplets area fraction (%), (**I**) mean multilocular lipid droplet size (µm^2), (**J**) mean unilocular lipid droplet size (µm^2). Each point is a single replicate from an ROI with an area of 333 x 333 um^2. *P < 0.05; **P < 0.01; ***P < 0.001.

Supplemental Figure 5

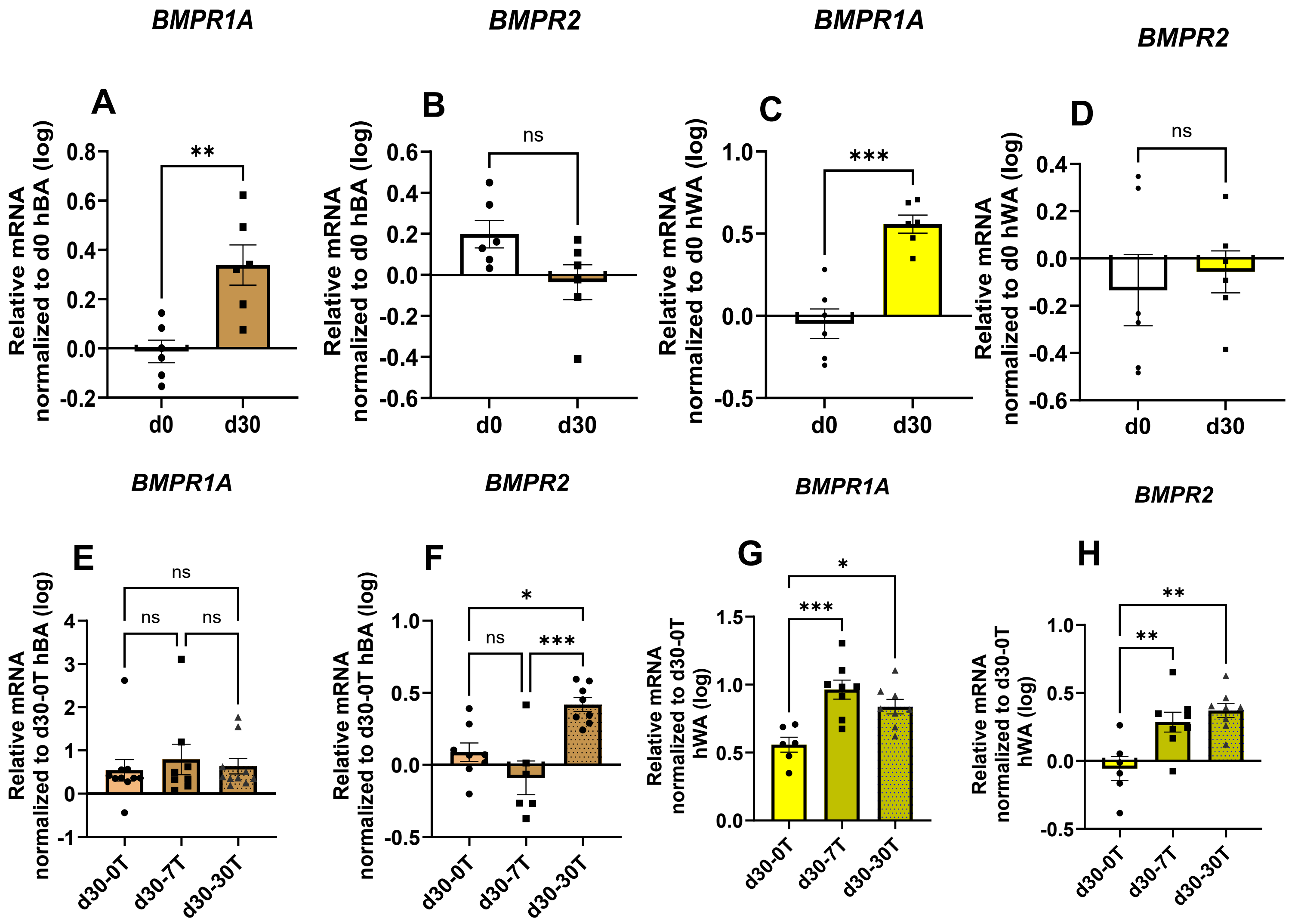

Supplemental Fig. 5. qPCR analysis of hBA adipocytes at 0 and 30 days of differentiation without BMP7 treatment (light brown bars), 30 days of differentiation with 7 days of BMP7 treatment (dark brown bars), and 30 days of differentiation with 30 days of BMP7 treatment (checkered dark brown bars). qPCR analysis of hWA adipocytes at 0 and 30 days of differentiation without BMP7 treatment (yellow bars), 30 days of differentiation with 7 days of BMP7 treatment (dark yellow bars), and 30 days of differentiation with 30 days of BMP7 treatment (checkered dark yellow bars). The mRNA expression profiles of **(A-B)** d0 undifferentiated and d30 differentiated hBA, **(C-D)** d0 undifferentiated and d30 differentiated hWA, **(E-F)** d30 hBA normalized to d30 negative control, **(G-H)** d30 hWA normalized to d30 to negative control. *P < 0.05; **P < 0.01; ***P < 0.001; ****P < 0.0001 (n=6 replicates). Each data point shown represents a biological replicate.

Supplemental Figure 6

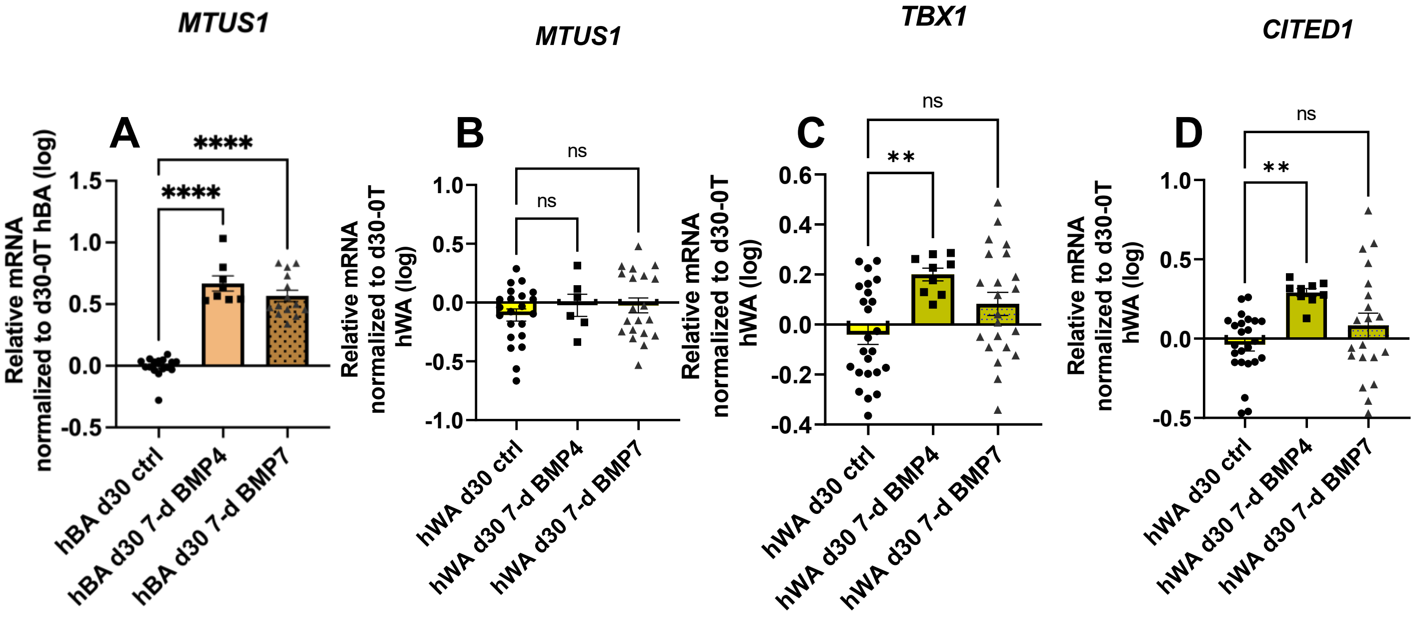

Supplemental Fig. 6. qPCR analysis of hBA and hWA adipocytes at 30 days of differentiation with 0 days of BMP treatment (dark brown bars, hBA or bright yellow bar, hWA), 30 days of differentiation with BMP4 treatment (light brown bars, hBA or dark yellow bars, hWA), and 30 days of differentiation with 7 days of BMP7 treatment (checkered dark brown bars, hBA or checkered dark yellow bars, hWA). The mRNA expression profiles of **(A-B)** brown adipocyte marker and **(C-D)** beige adipocyte markers**,** *P < 0.05; **P < 0.01; ***P < 0.001; ****P < 0.0001 (n=8-24 replicates). Each data point shown represents a biological replicate.

Supplemental Figure 7

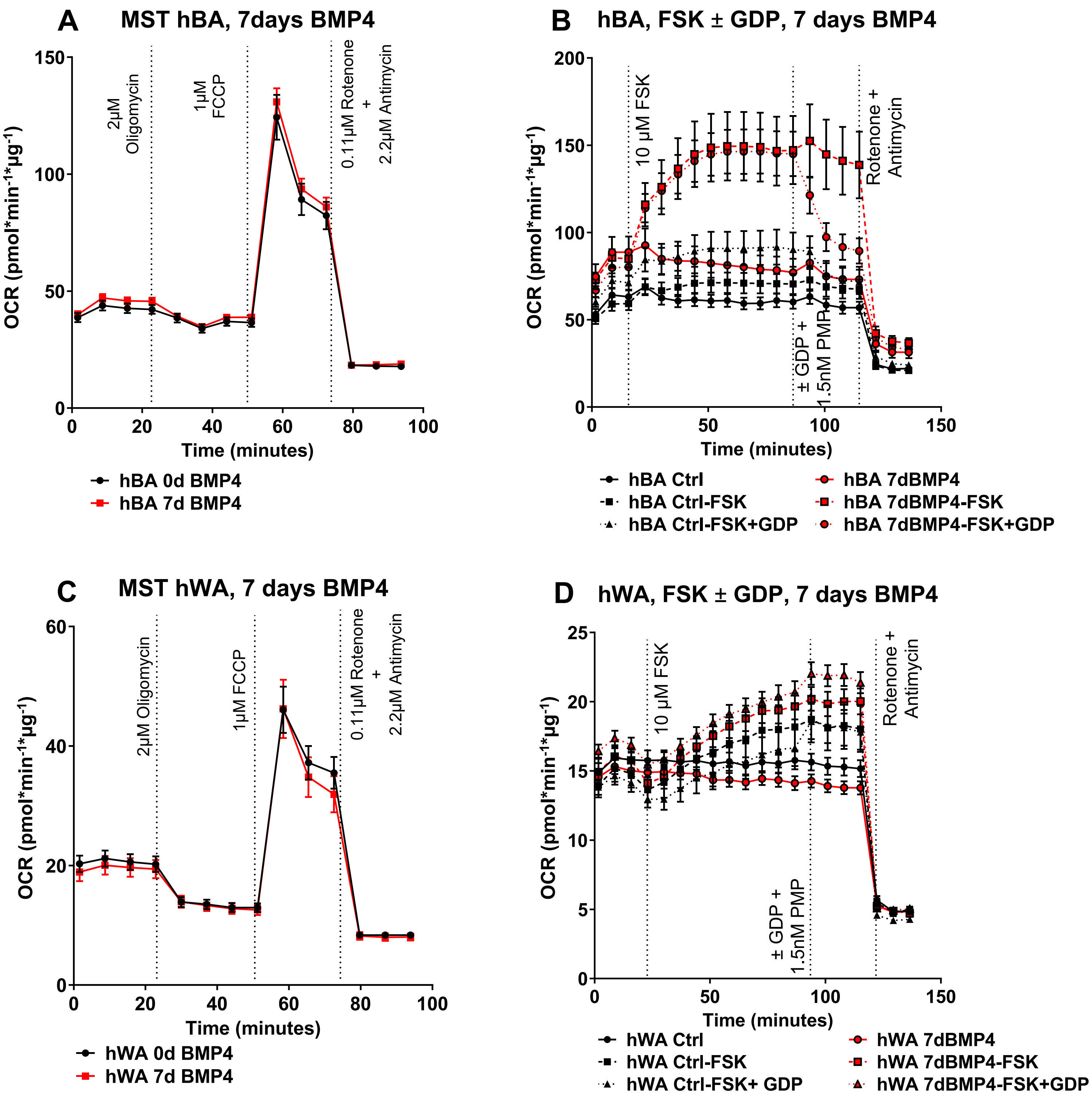

Supplemental Fig. 7. 30-day BMP4 treatment affected cellular bioenergetics in hBA and hWA. Mitochondrial stress test with oxygen consumption rate (OCR) tracings in **(A & C)** hBA and hWA with or without FSK and GDP (n=35-40 replicates for each treatment group). hBA or hWA treated with 0d BMP4 in black line and 30d BMP4 treated hBA or hWA in red line. **(B & D)** OCR after addition of forskolin (Fsk) ± GDP in hBA or hWA with 30d BMP4. (n=35-40 replicates for each treatment group). hBA or hWA control in black lines and hBA or hWA with BMP4 in red lines.

Supplemental Figure 8

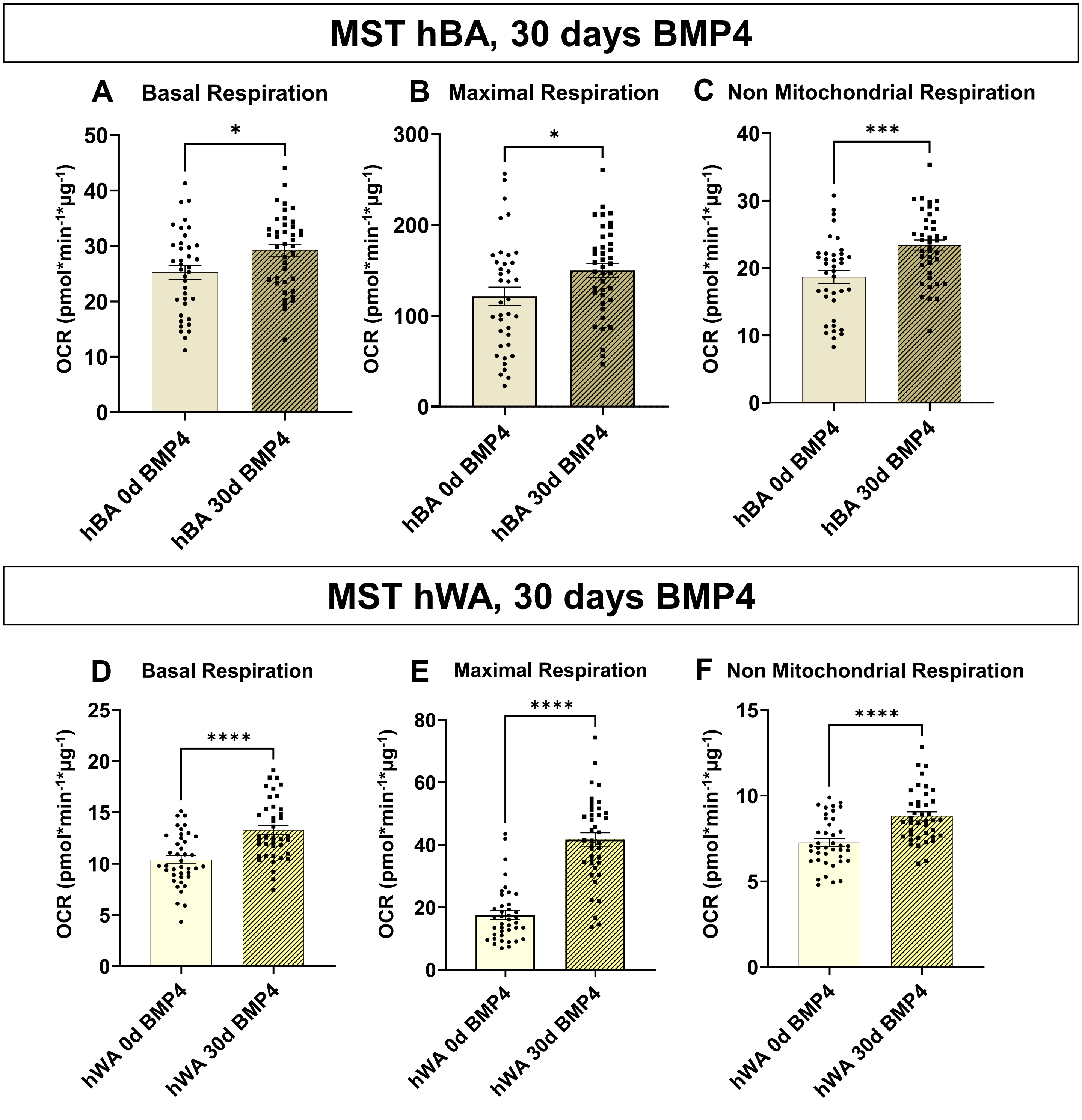

Supplemental Fig. 8. Mitochondrial stress test (MST): quantified respiratory profile of hBA showing **(A)** basal respiration, **(B)** maximal respiration, and **(C)** non-mitochondrial respiration. (n=35-40 replicates for each treatment group). hBA with no BMP4 in light brown bar and hBA treated with 30 days BMP4 in dashed dark bar. Quantified respiratory profile of hWA cells showing **(D)** basal respiration, **(E)** maximal respiration, and **(F)** non-mitochondrial respiration. (n=35-40 replicates for each treatment group). hWA treated with the standard differentiation protocol in light yellow bar and hWA treated with 30 days BMP4 in dashed yellow bar.

Supplemental Figure 9

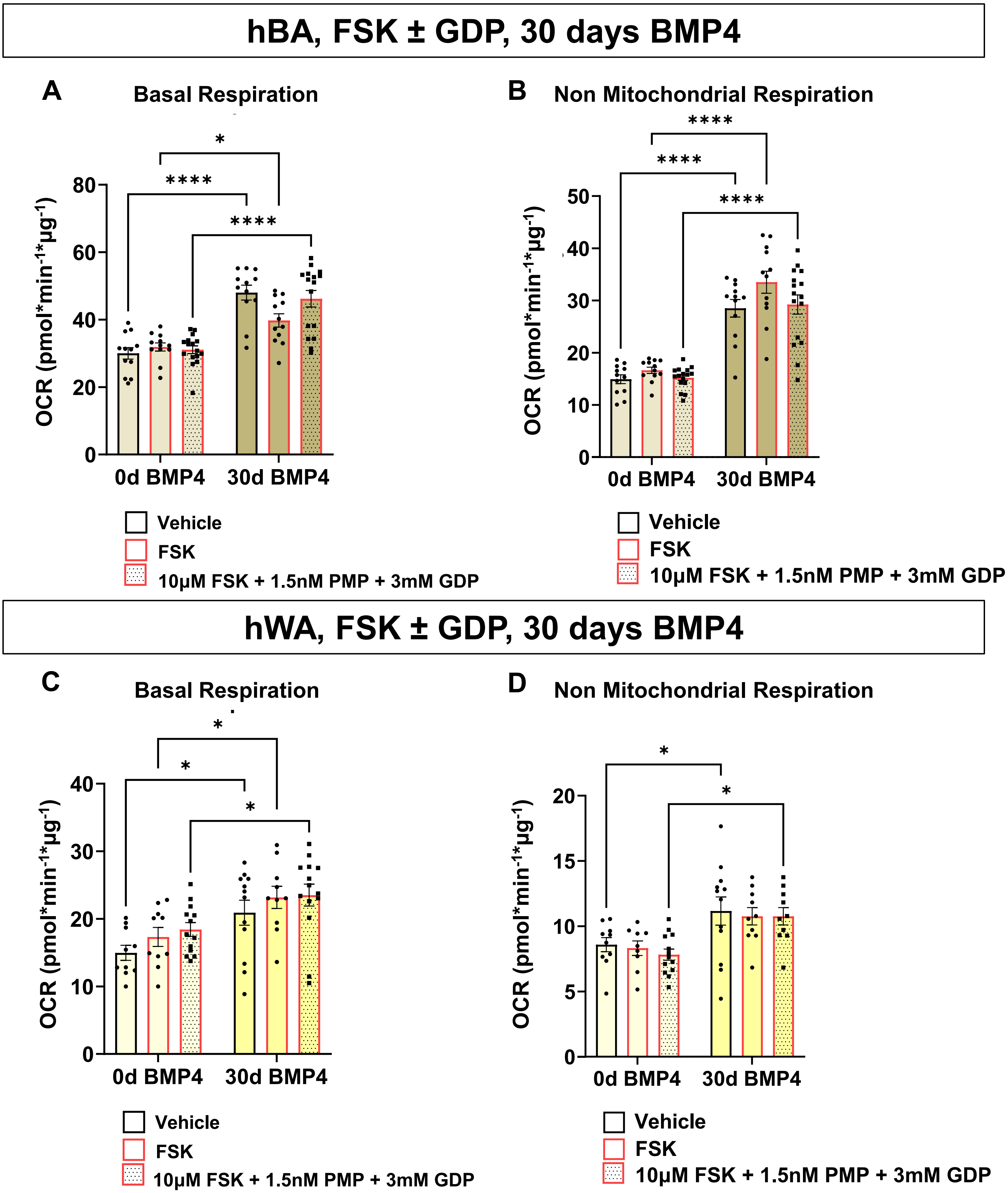

Supplemental Fig. 9. Quantified respiratory profile of hBA following addition of FSK with or without GDP showing **(A)** basal respiration and **(B)** non-mitochondrial respiration. (n=12-16) replicates for each treatment group). Quantified respiratory profile of hWA cells showing **(C)** basal respiration and **(D)** non-mitochondrial respiration. (n=12-16 replicates for each treatment group).

Supplemental Figure 10

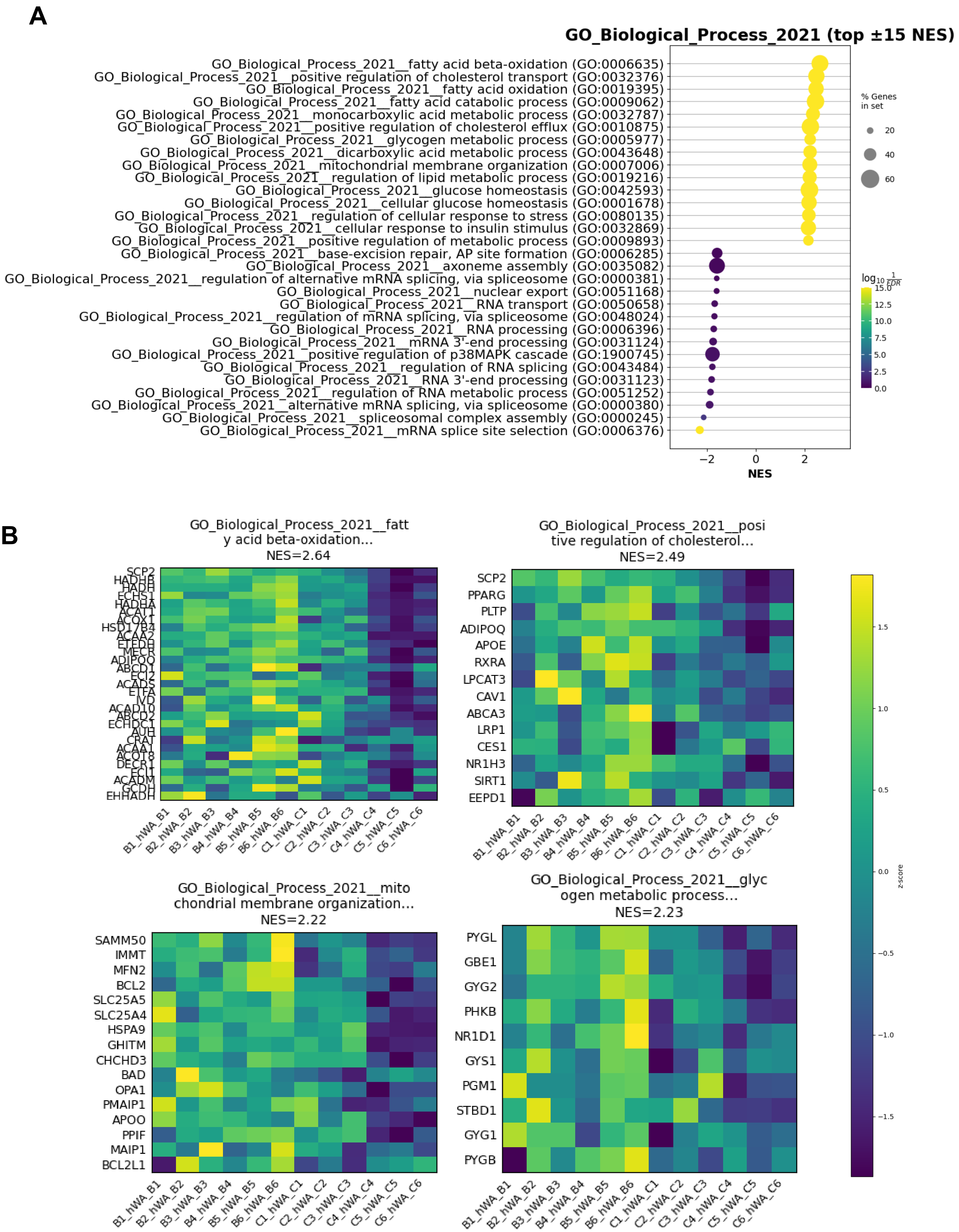

Supplemental Fig. 10. RNA-seq comparison of hWA differentiated without or with BMP7 (d0-d7). (**A**) Bubble plot showing representative pathways from gene ontology (GO) analysis of enriched genes. (**B**) Heatmaps of the top 25 genes relevant to the individual GO terms. Dataset identifiers on the x-axis indicate either hWA differentiated without (starting with"C's") or with BMP7 (d0-d7) (starting with "B's").

Supplemental Figure 11

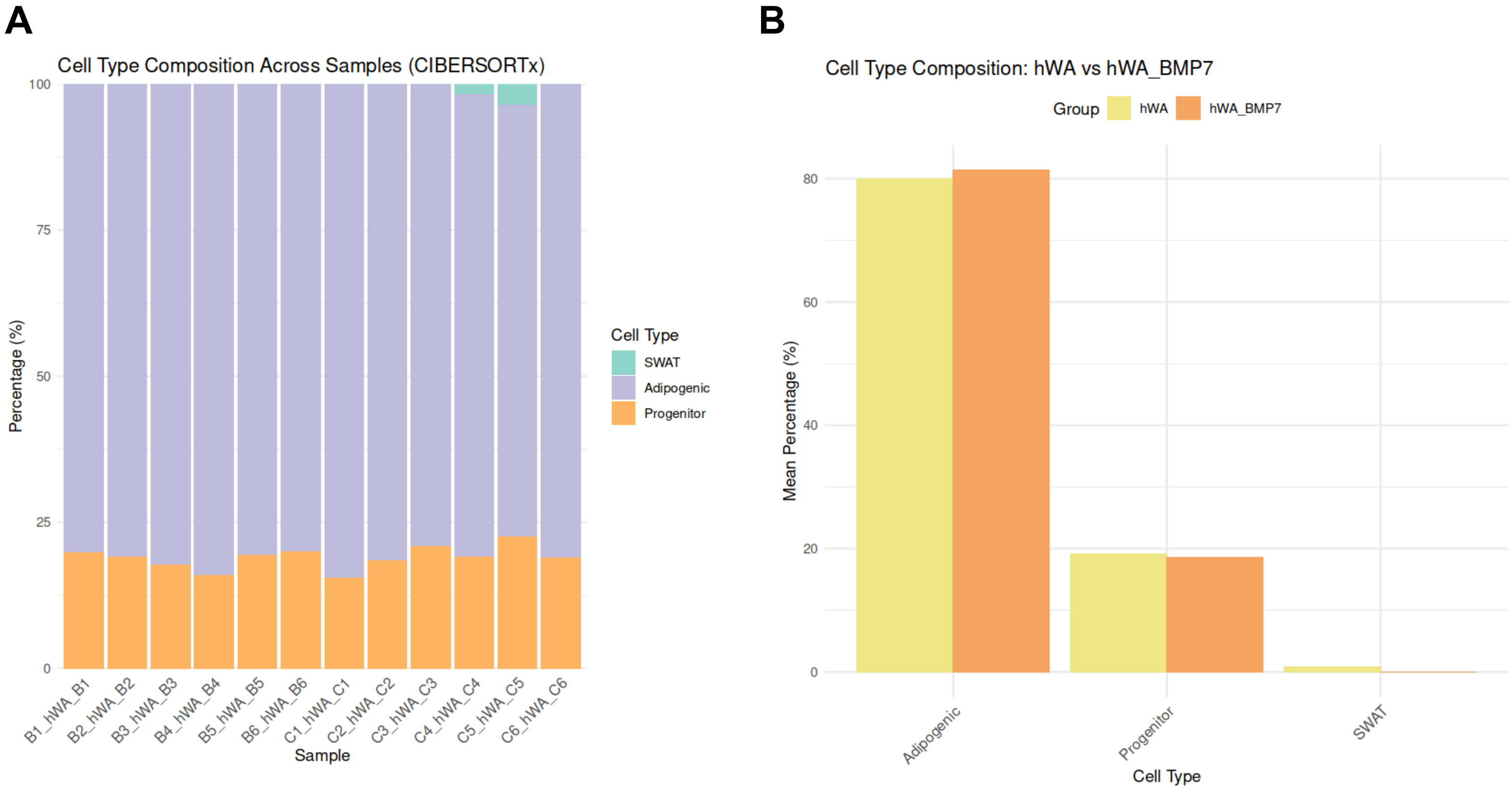

Supplemental Fig. 11. Deconvolution of bulk RNA-seq data of hWA cells differentiated without or with BMP7 (d0-d7), six each, using Cibersortx to determine the presence of structural Wnt-regulated adipose tissue-resident (SWAT), adipogenic, and progenitor cell types. (**A**) Shown are the relative proportions of the three cell types in each of the bulk RNA-seq datasets based on the Cibersortx algorithm. X-axis labels starting with ”C” indicate hWA samples differentiated without BMP7 and labels starting with “B” indicate hWA samples differentiated with BMP7 (d0-7). (**B**) Comparative visualization of relative proportions of the three cell types between hWA samples differentiated without BMP7 and hWA samples differentiated with BMP7 (d0-7).

Supplemental Figure 12

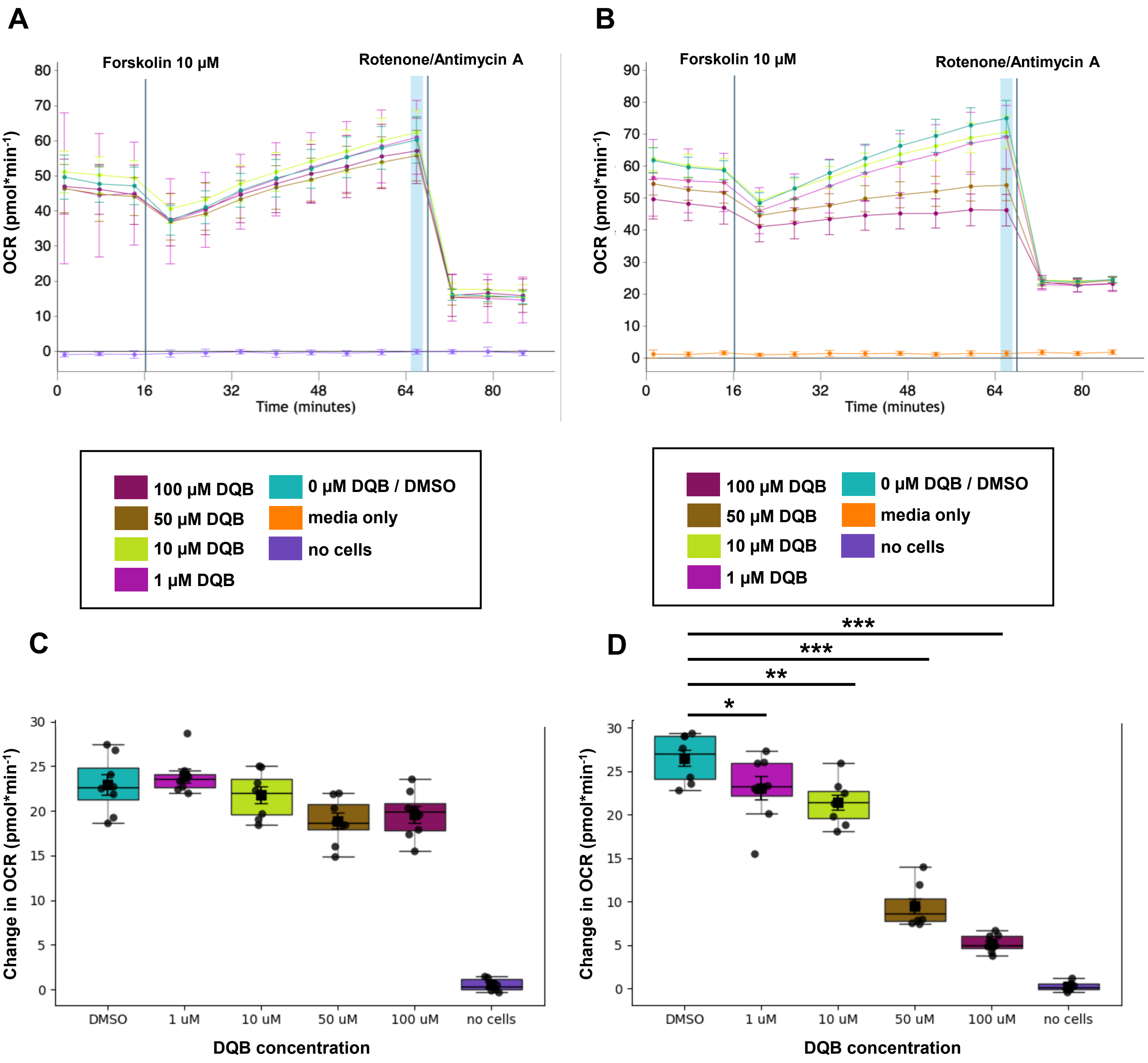

Supplemental Fig. 12. hWA were differentiated (**A**) without or (**B**) with BMP7 (d0-d7) ("tan cells") and on Day 30 treated with different doses of the tissue non-specific alkaline phosphatase (TNAP) inhibitor 2,5-Dimethoxy-N-(quinolin-3-yl)benzenesulfonamide (DQB) for 30 minutes prior to addition of 10 µM forskolin. Oxygen consumption rates (OCR) were measured over 44 minutes followed by addition of rotenone (0.11 µM) and antimycin A (2.2 µM), (n=8 independent replicates for each treatment group). Dose response of DQB for hWA differentiated **(C)** without or **(D)** with BMP7 for 7 days. Box plots depict mean ± SEM. * $P < 0.05$, ** $P < 0.01$, *** $P < 0.001$.
