# Supplementary material for: Bone morphogenetic proteins 4 and 7 increase human white and brown adipocyte thermogenic capacity
Source: JCI Insight. 2026 Mar 12;11(8):e194140. doi: 10.1172/jci.insight.194140 (PMC13135406; doi:10.1172/jci.insight.194140)
Supplement: Unedited blot and gel images [file jciinsight-11-194140-s128.pdf]

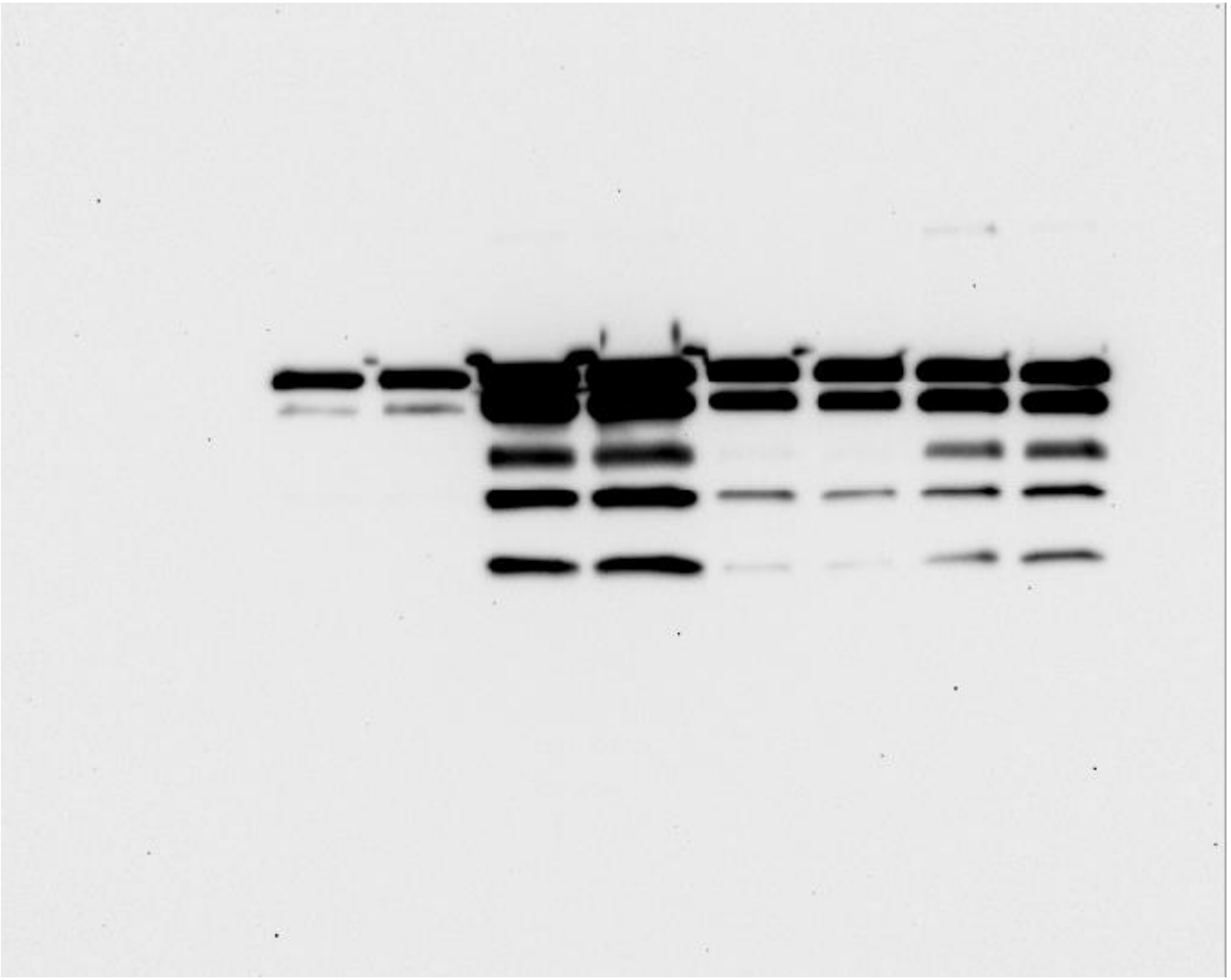

hBA BMP7 OxPhos

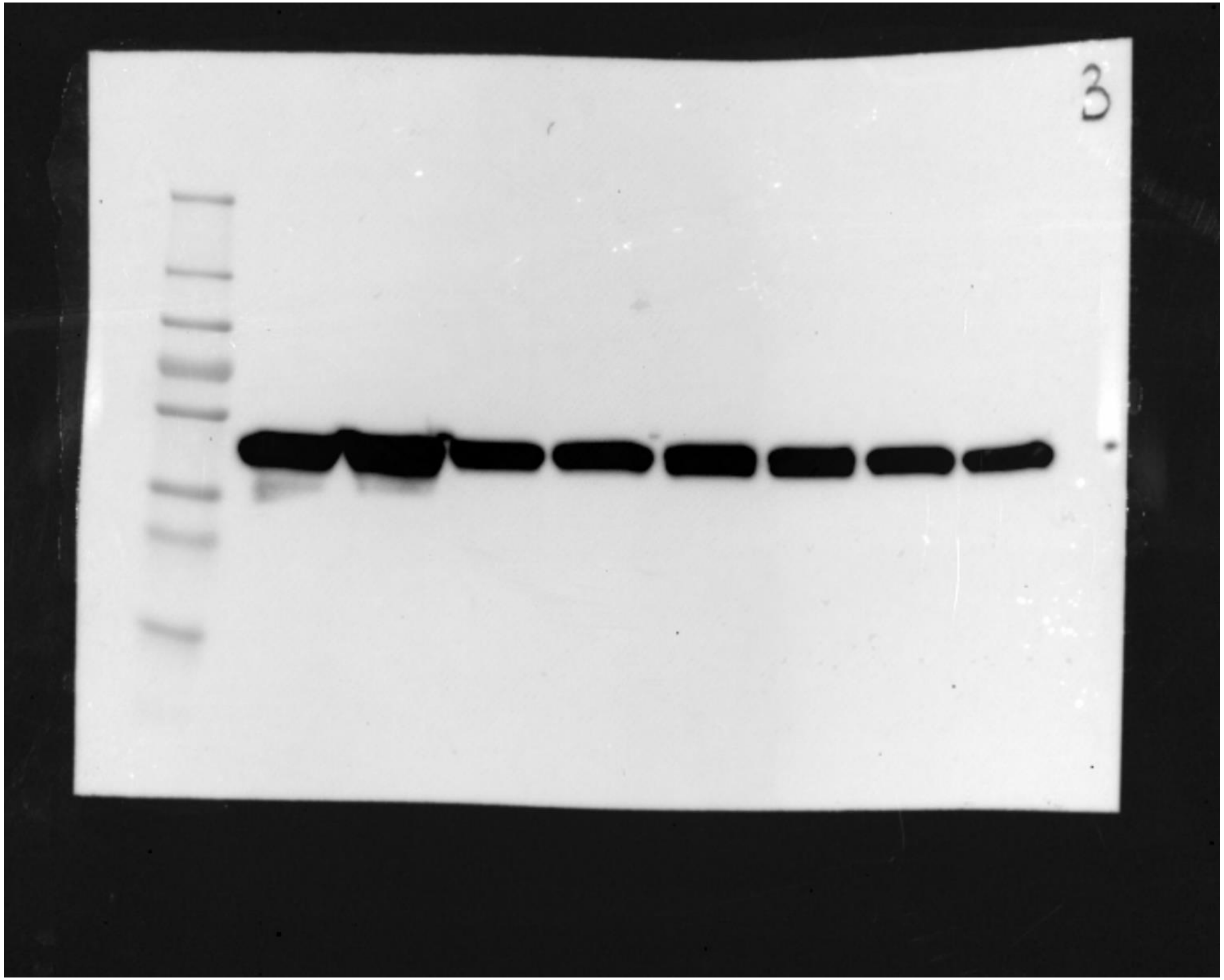

hBA BMP7 Actin

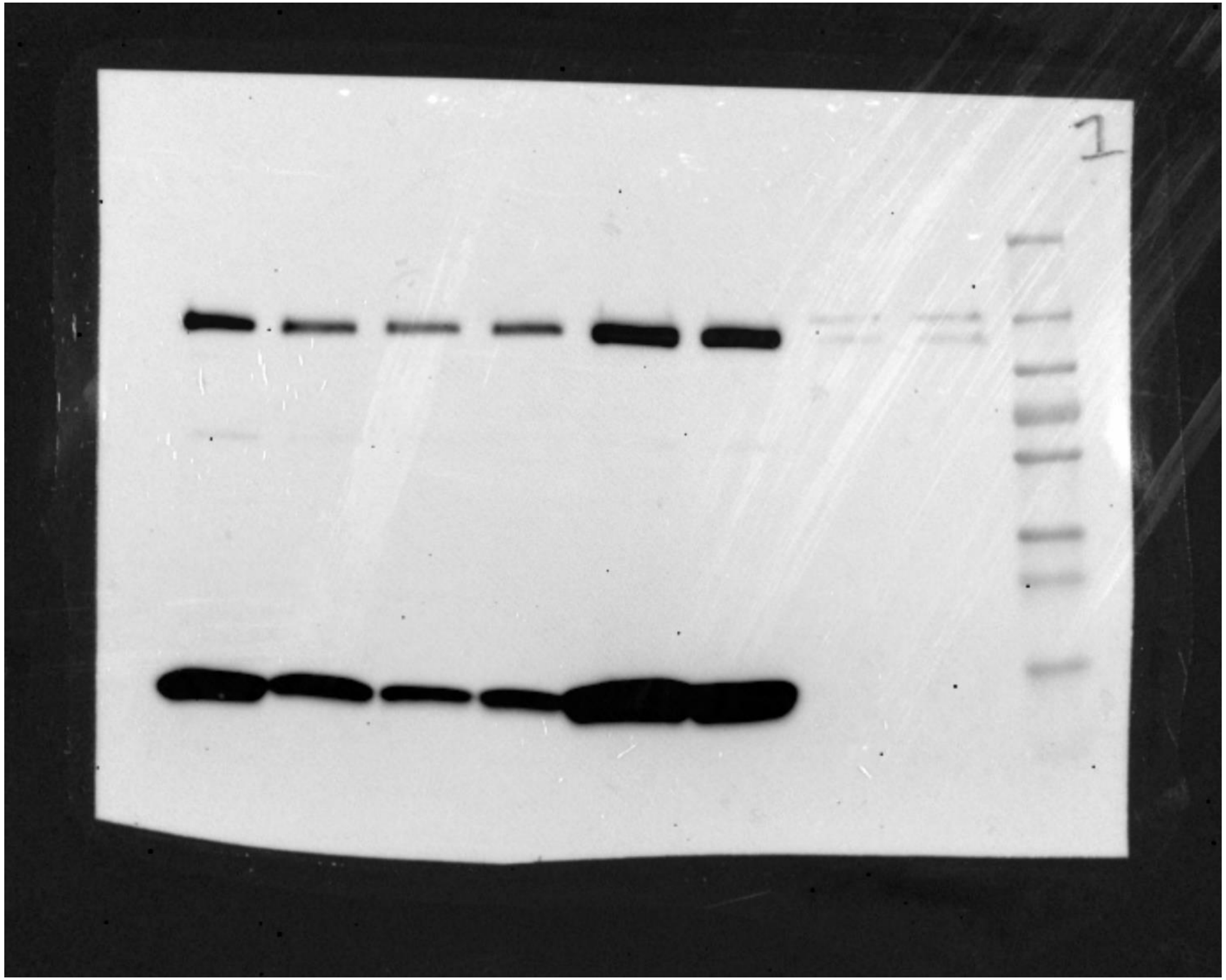

hBA BMP7 PGC1a

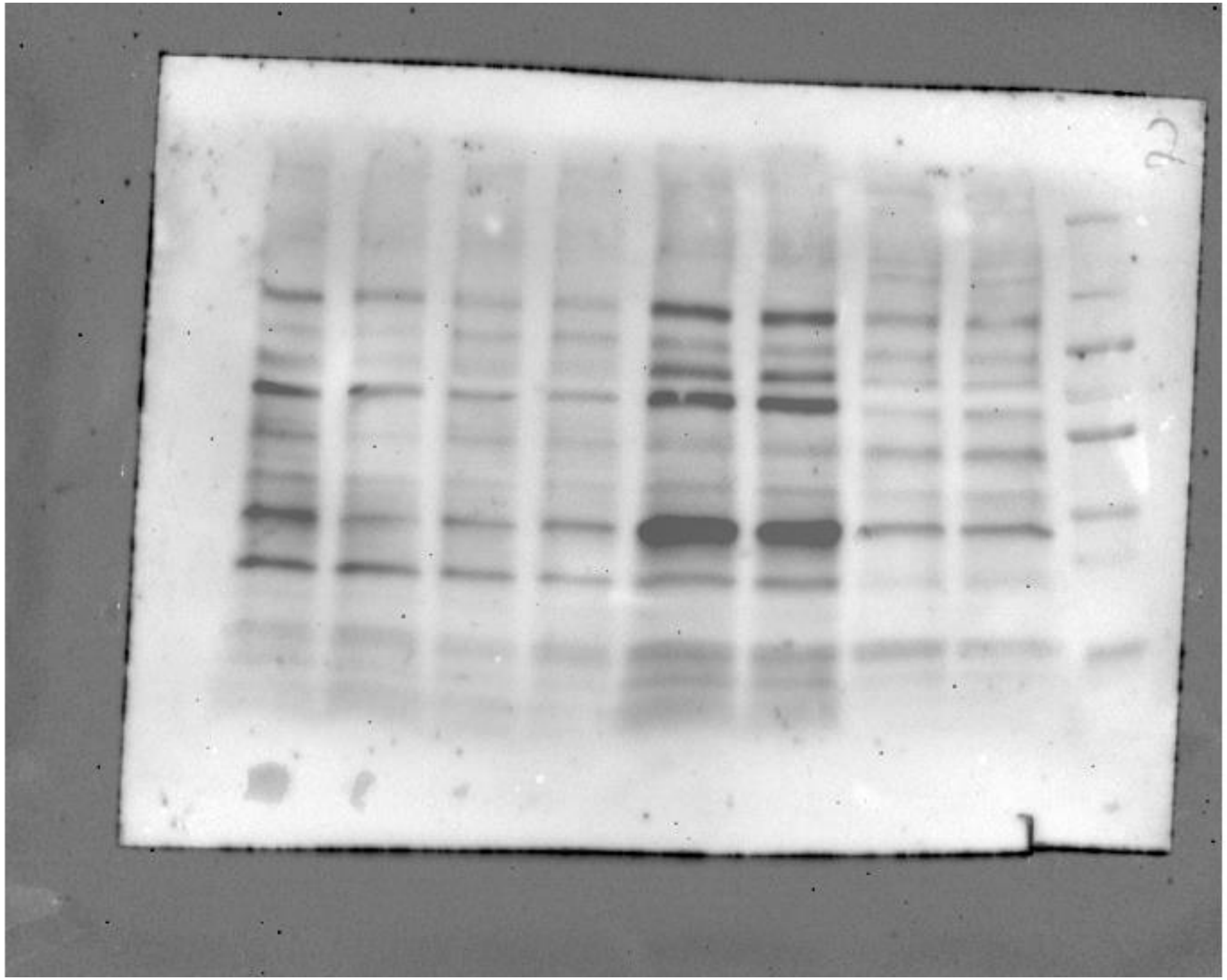

hBA BMP7 UCP1

2023-05-08 12hr 24min 33sec+2023-05-08 12hr 20min 19sec

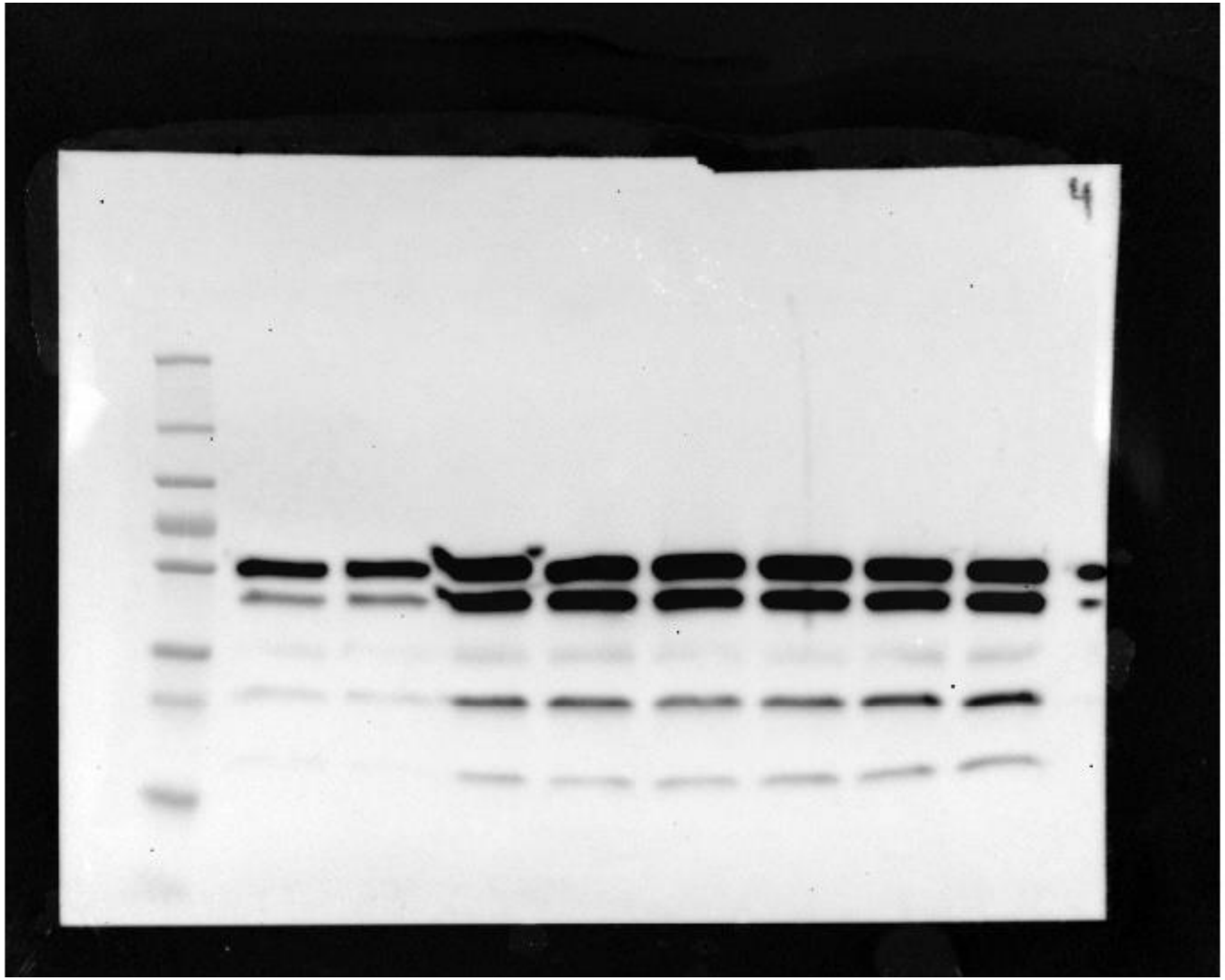

hWA BMP7 Oxphos

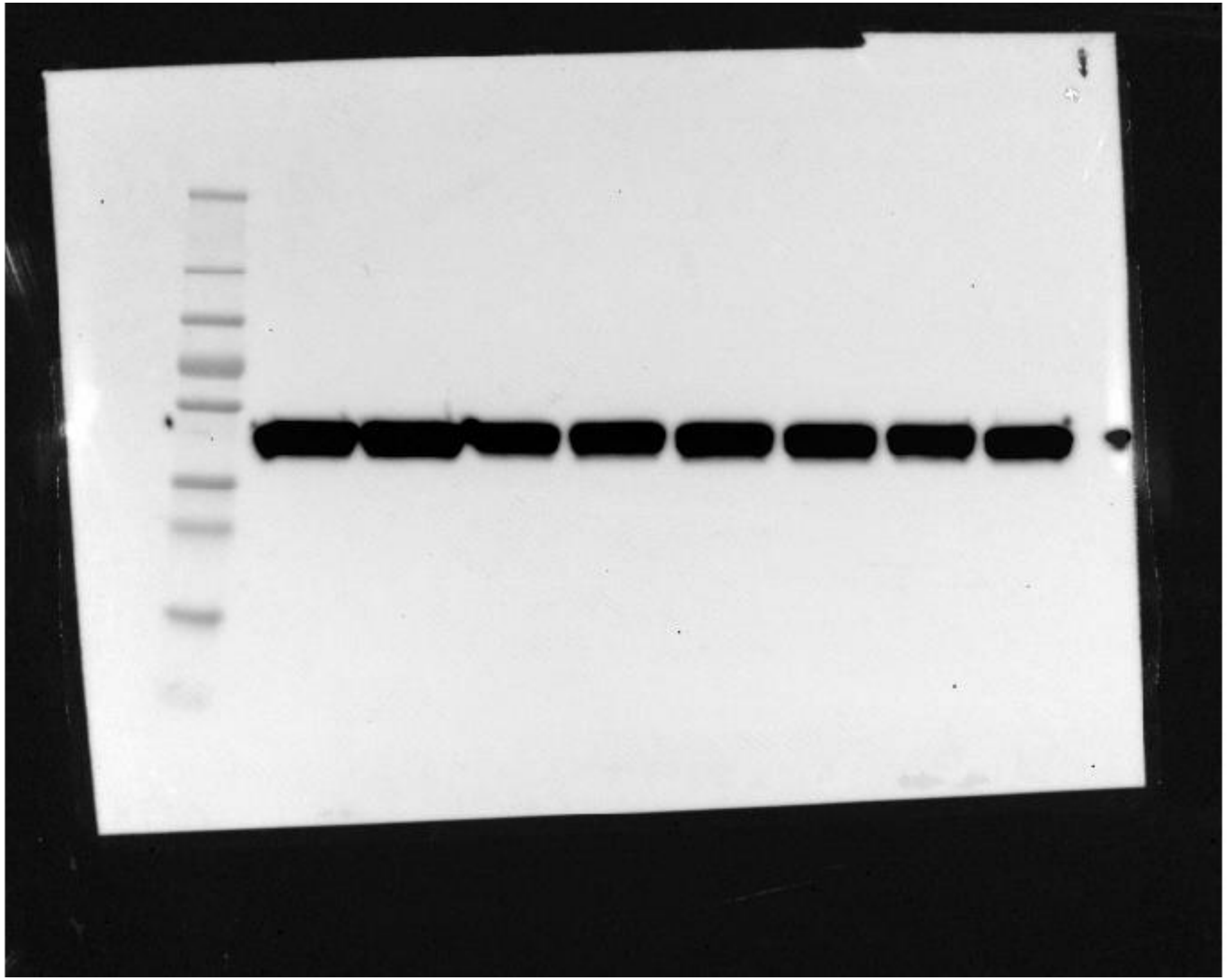

hWA BMP7 Actin

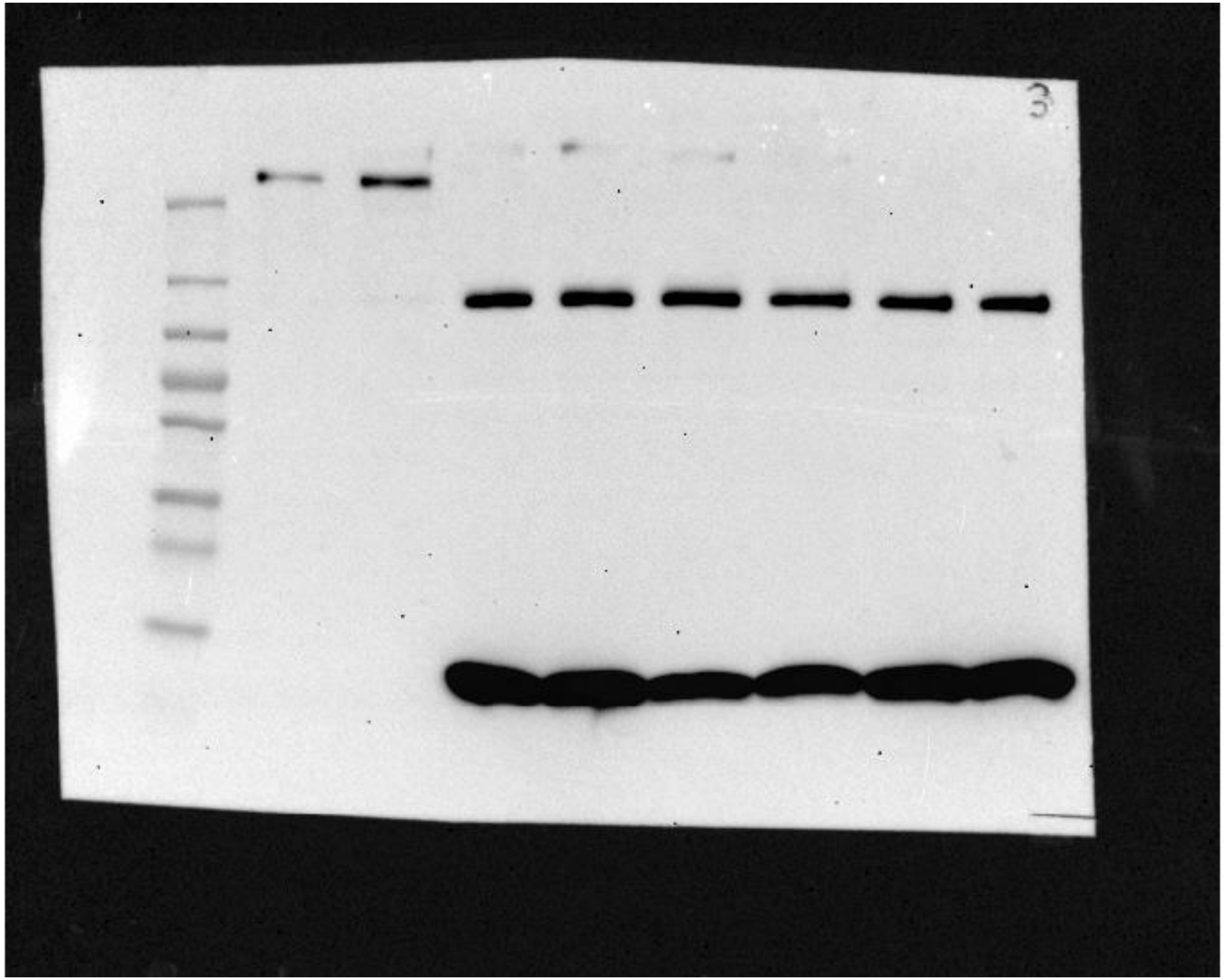

hWA BMP7 PGC1a

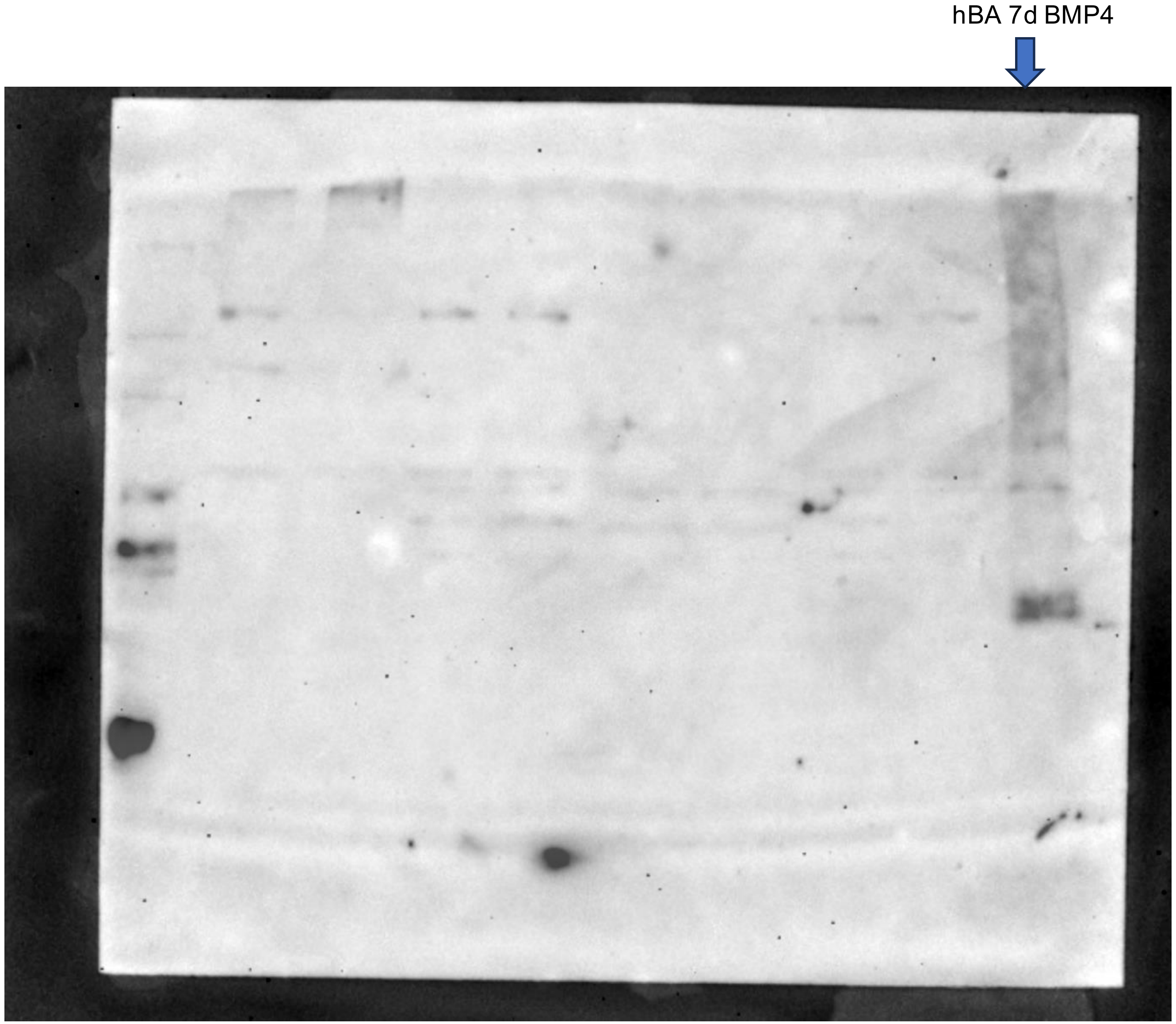

hWA BMP7 UCP1

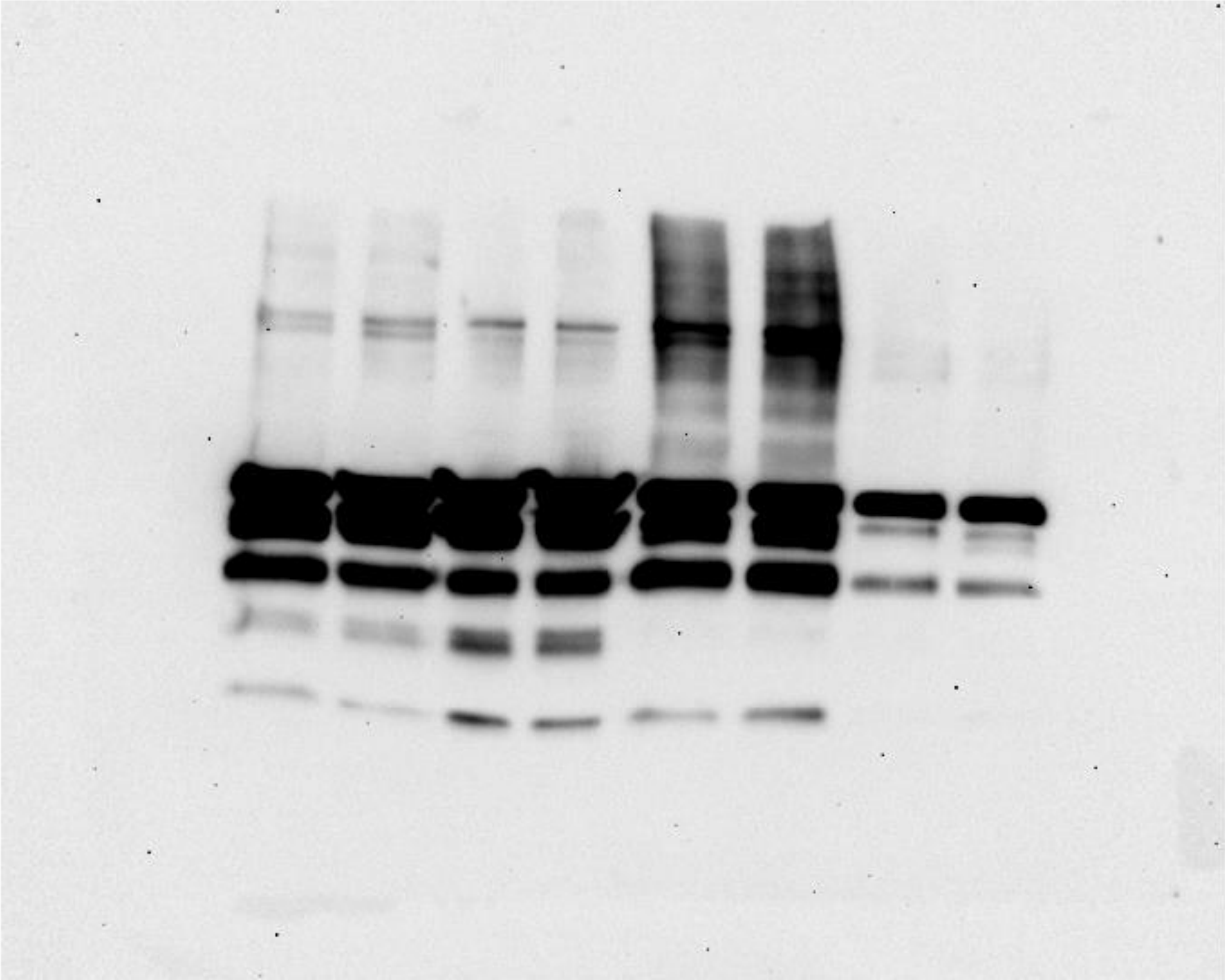

hBA BMP4 Oxphos

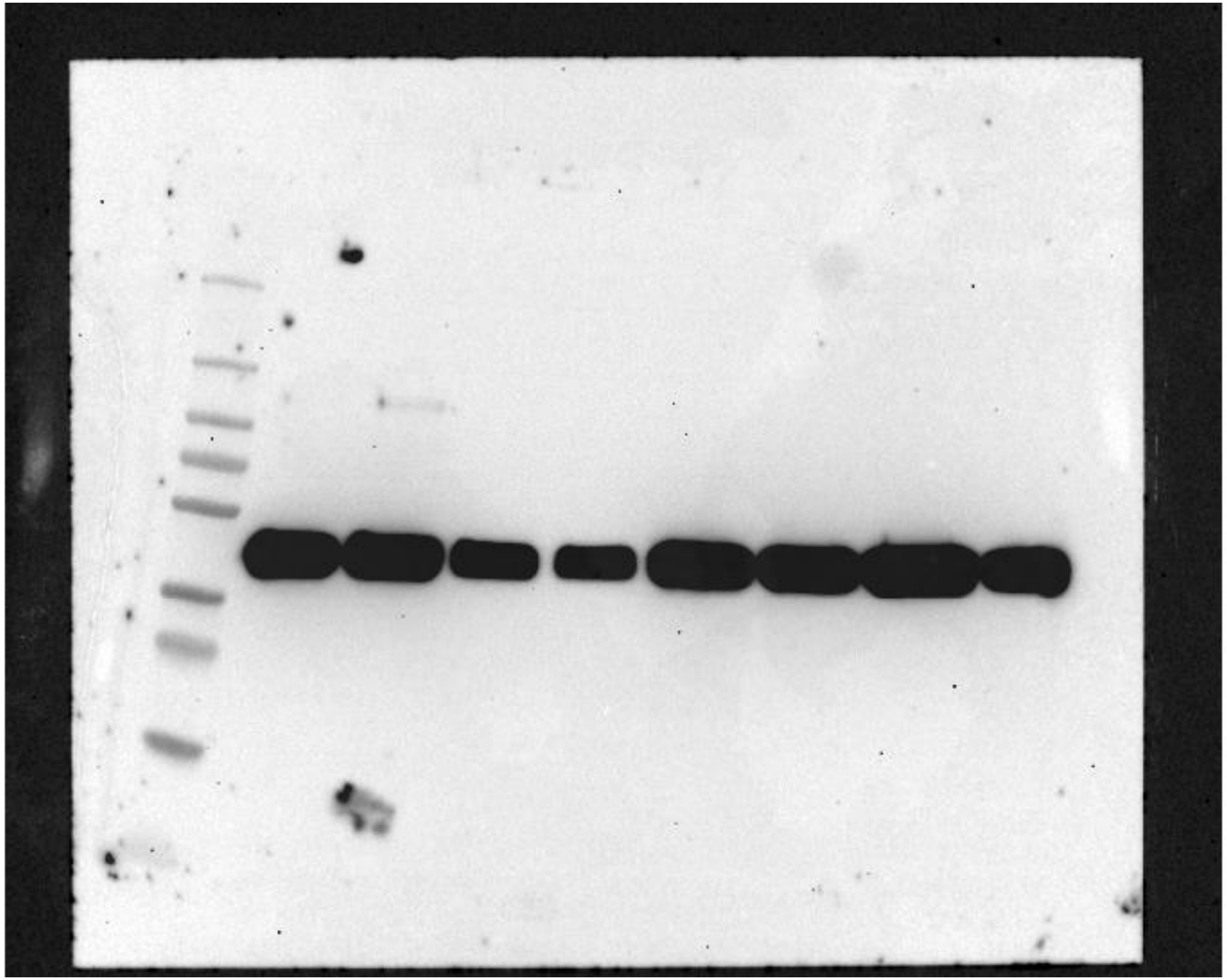

hBA BMP4 Actin

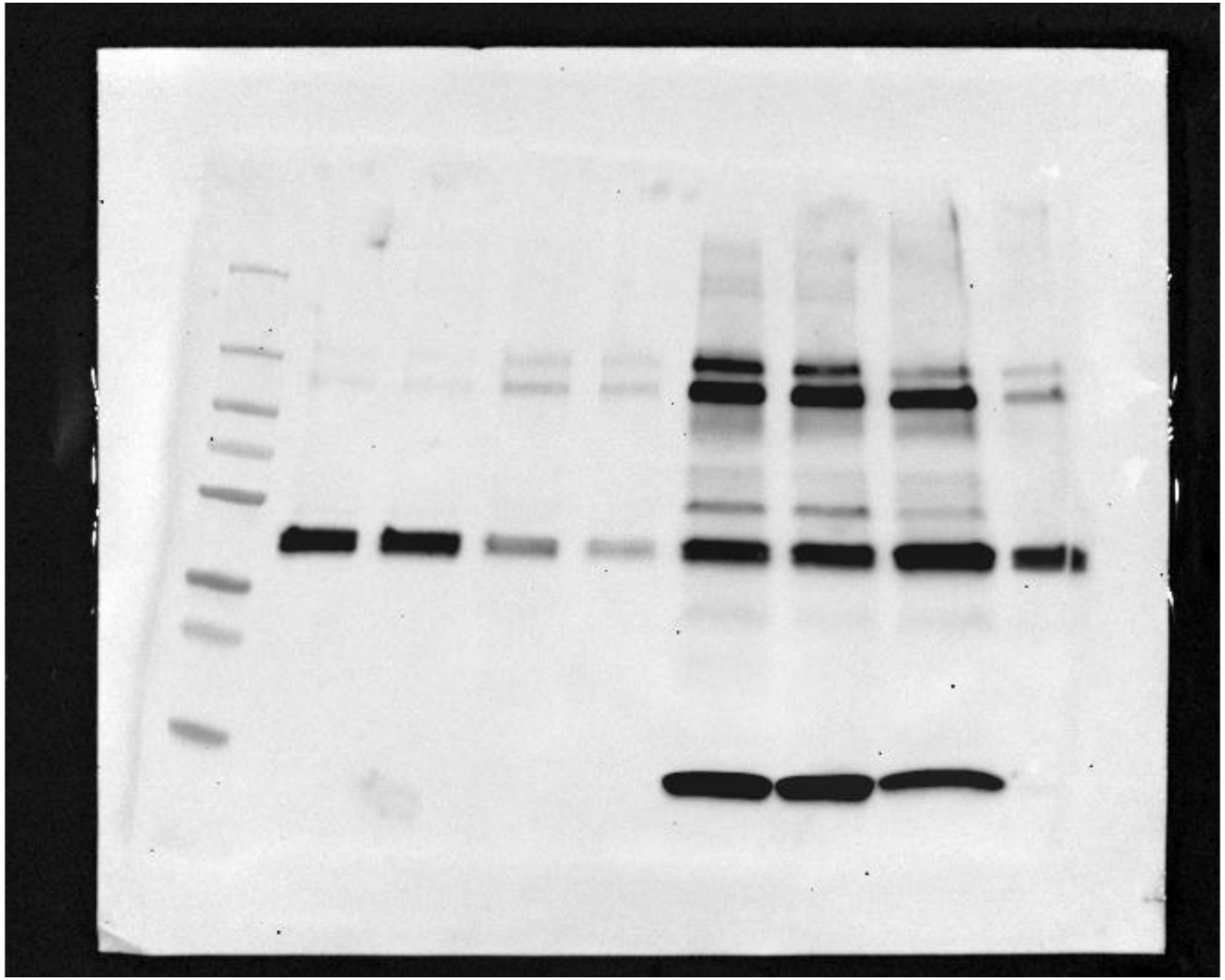

hBA BMP4 PGC1a

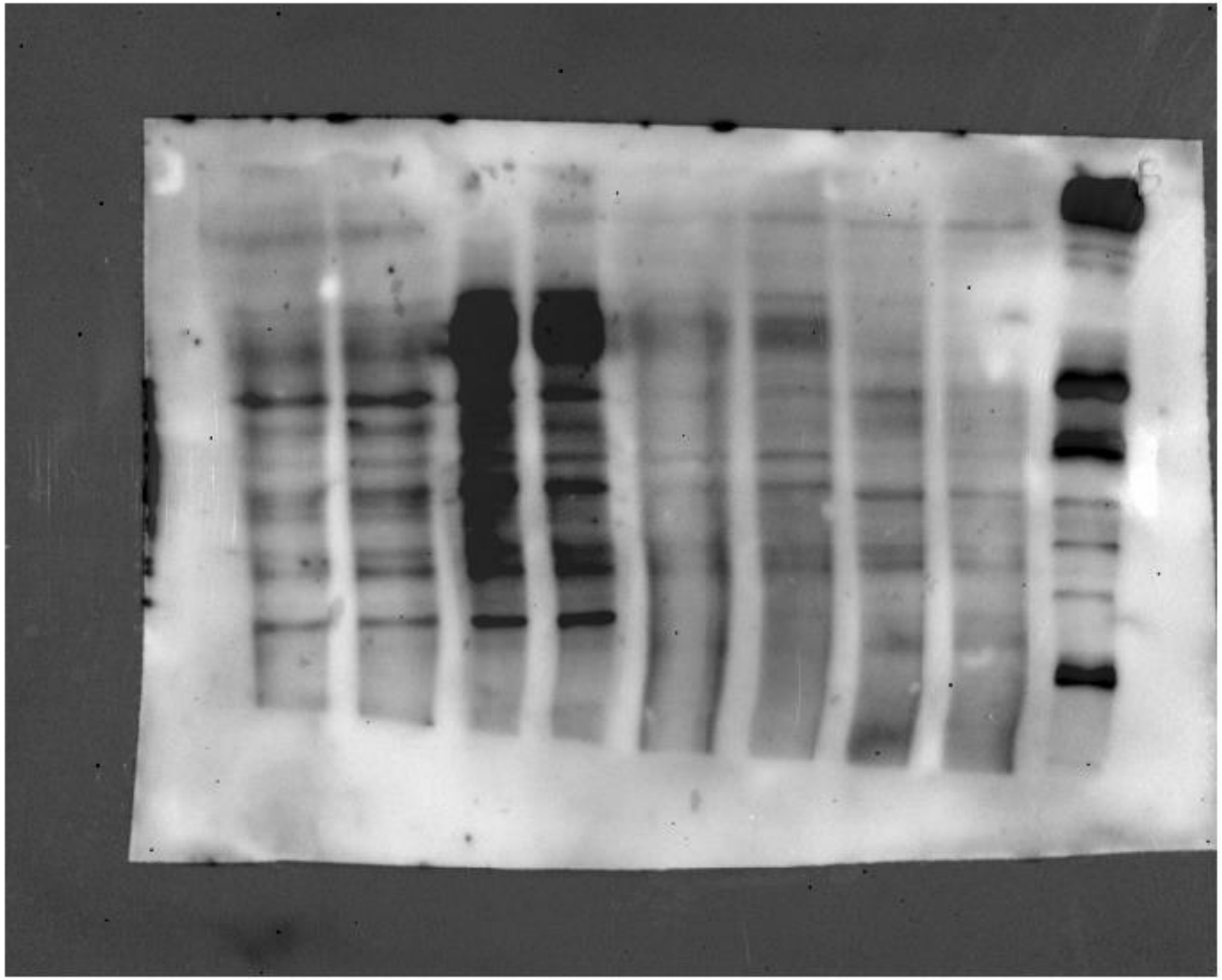

hBA BMP4 UCP1

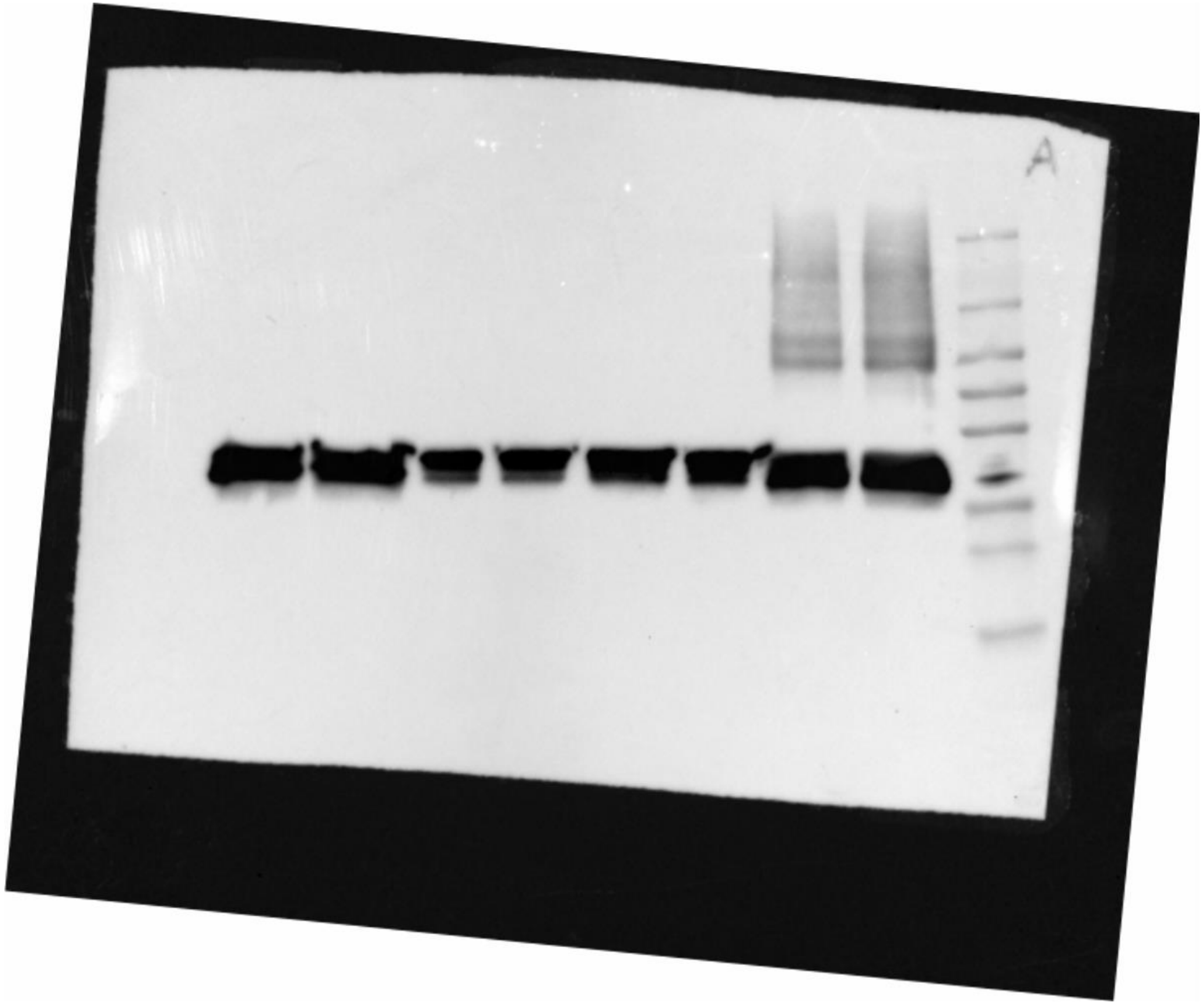

hWA BMP4 Actin

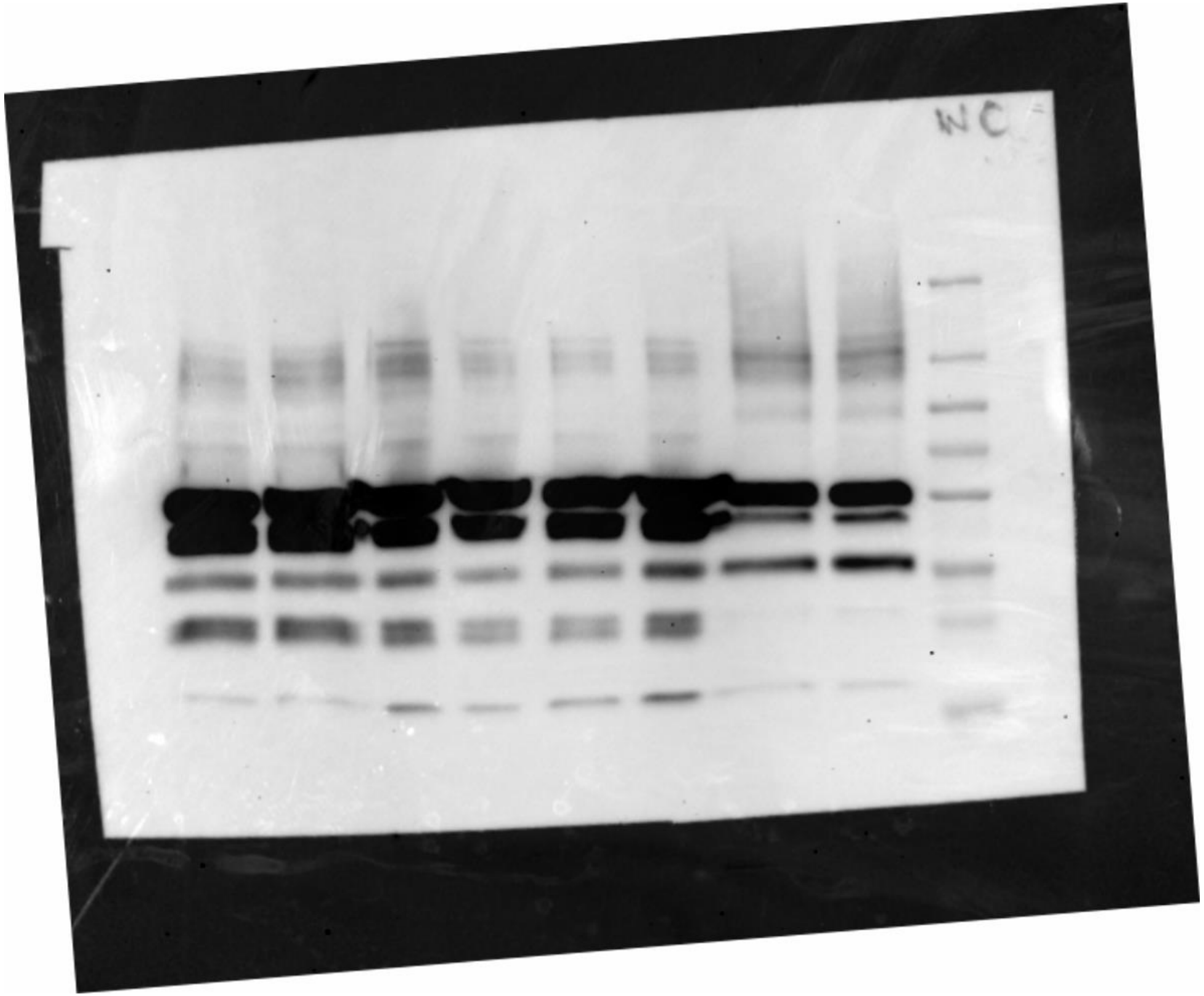

hWA BMP4 Oxphos

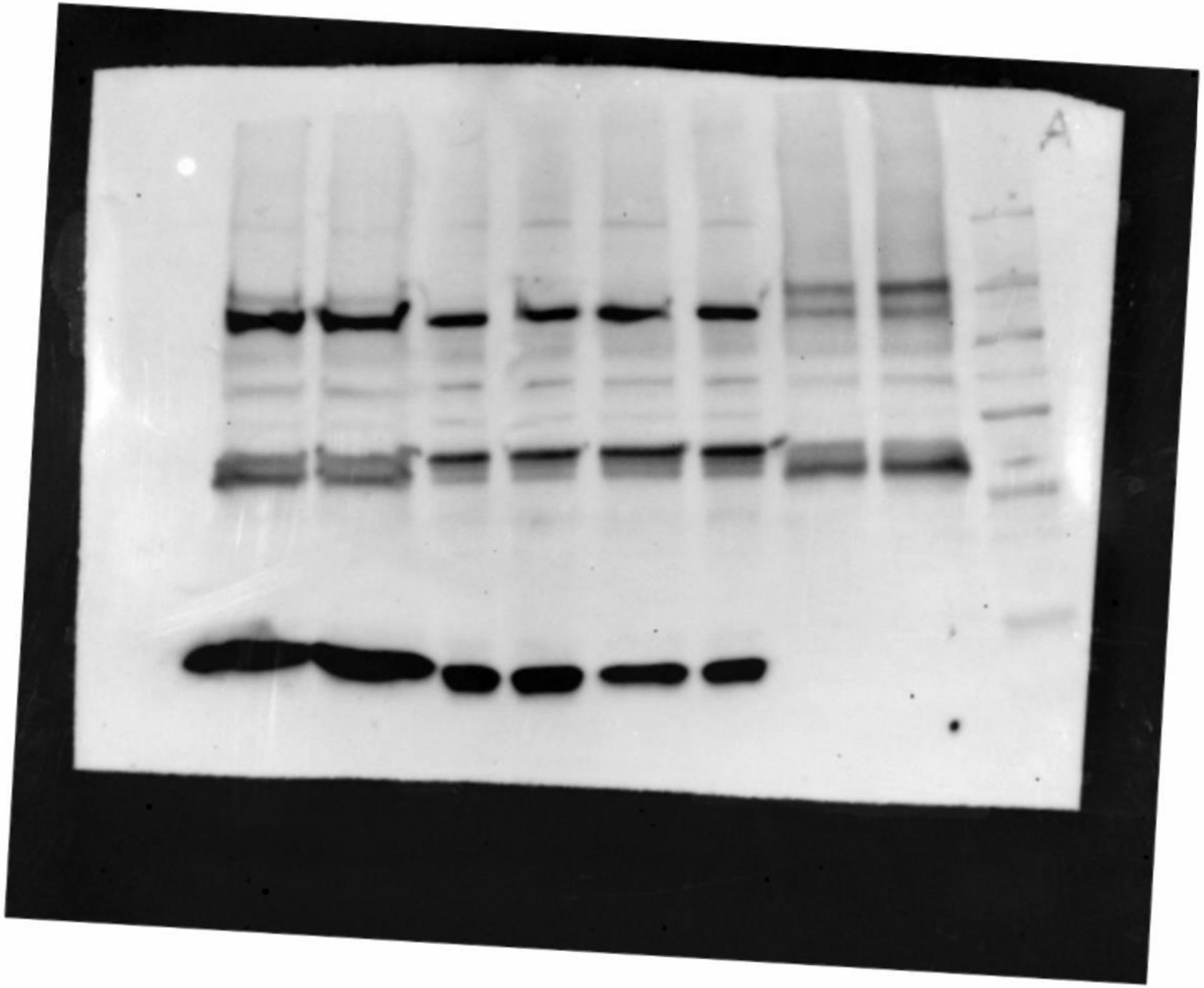

hWA BMP4 PGC1a

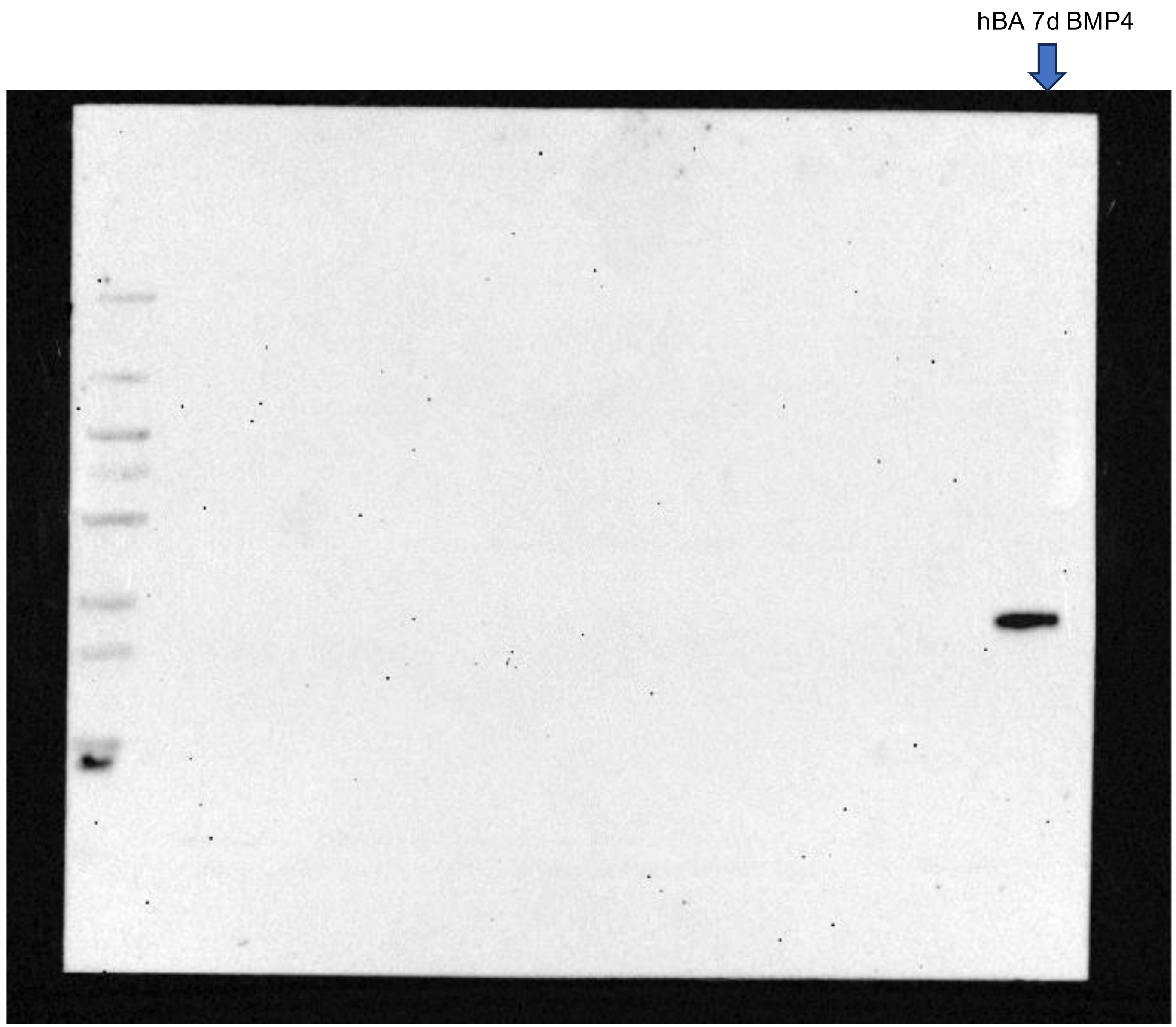

hWA BMP4 UCP1
